# Association of per- and polyfluoroalkyl substances with gout risk: a cross-sectional analysis of NHANES 2007–2018 data emphasizing mixture effects

**DOI:** 10.3389/fpubh.2025.1484663

**Published:** 2025-02-10

**Authors:** Haixin Feng, Siran Li, Shiqing Huang, Linxi He, Ruihao Huang, Renhuizi Wei, Xin Peng, Haiyi Yan, Chongxiang Xiong, Bingsong Zhang

**Affiliations:** ^1^Department of Epidemiology and Biostatistics, School of Public Health, Guangdong Medical University, Dongguan, China; ^2^Office of Quality Management, Hospital of Huangjiang Dongguan, Dongguan, China; ^3^Department of Biostatistics, School of Public Health, Southern Medical University, Guangzhou, China; ^4^Department of Nephrology, The First Dongguan Affiliated Hospital of Guangdong Medical University, Dongguan, China

**Keywords:** mixture exposure, PFAS (per- and polyfluoroalkyl substances), gout, WQS regression, logistic regression

## Abstract

**Objective:**

This study examined associations between serum concentrations of per- and polyfluoroalkyl substances (PFASs) and gout risk in the U.S. adult population using the National Health and Nutrition Examination Survey (NHANES) 2007–2018 data. And assessing the potential intermediary effect of uric acid.

**Methods:**

The study included 8,494 participants, with 385 having gout. Four PFAS compounds (PFOA, PFOS, PFHxS, PFNA) were measured. PFOS is the most prevalent PFAS in the environment, biota, and human tissues. It is rapidly absorbed and accumulates in the liver, kidneys, and blood, binding to serum albumin and low-density lipoprotein. PFOA is highly persistent in the body, mainly accumulating in the kidneys and liver through enterohepatic circulation, posing risks due to its difficulty in metabolism and excretion. PFHxS has the longest metabolic half-life in humans (7.3 years) and bioaccumulates in the endocrine, immune, nervous, and reproductive systems. PFNA is the second most detected PFAS in human serum after PFOS. It is more likely to accumulate and express toxicity in the reproductive organs, liver, and immune system compared to PFOS and PFOA. Multivariate logistic regression and weighted quantile sum regression were used to assess individual and mixture effects. Mediation analysis was conducted to estimate effect of uric acid.

**Results:**

In fully adjusted model, the associations were nonsignificant, with PFOA showing a marginally positive association. Mixture analysis revealed a significant positive association with gout risk across all models. PFOS was the largest contributor to the mixture effect. Stronger associations were observed in old people and females. Sensitivity analyses confirmed the robustness of these findings. Mediation analysis indicated significant intermediary effect of uric acid in the associations of PFAS with risk of gout, with the mediated proportion ranging from 48 to 77%.

**Conclusion:**

This study provides evidence for a potential link between PFAS exposure and gout risk, particularly when considering mixtures. While associations with individual PFASs are largely explained by demographic and lifestyle factors, the persistent association of mixtures with gout risk highlights the importance of considering combined exposures in environmental health research. Uric acid level plays a crucial intermediary effect.

## 1 Introduction

PFASs are a class of synthetic chemicals characterized by their persistence in the environment and potential for bioaccumulation in living organisms (1). Since their introduction in the 1940s, PFASs have been widely used in various consumer and industrial applications because of their unique water- and oil-repellent properties (2). However, the widespread use and environmental persistence of PFASs have led to their ubiquitous presence in the environment and human body, raising significant concerns about their potential health impacts (3, 4). The health effects of PFAS exposure have been a subject of increasing research interest. Studies have linked PFAS exposure to various adverse health outcomes, including endocrine disruption, immunotoxicity, and metabolic disorders (5, 6). Of particular concern is the potential role of PFASs in the development of chronic diseases, including those related to metabolic dysfunction (7).

Gout, a form of inflammatory arthritis characterized by the deposition of monosodium urate crystals in joints and surrounding tissues, has emerged as a significant public health concern (8). The prevalence of gout has been increasing globally, with factors such as dietary changes, obesity, and an aging population contributing to this trend (9). While traditional risk factors for gout, including genetic predispositions and lifestyle factors, are well established, interest in understanding the potential role of environmental exposure in gout etiology is increasing (10). Recent epidemiological studies have suggested a potential link between environmental pollutants and the risk of gout. For example, exposure to lead and other heavy metals has been associated with elevated uric acid levels and increased gout risk (11). Given the widespread exposure to PFASs and their known effects on metabolic processes, there is a compelling rationale for investigating the potential association between PFAS exposure and gout risk (12).

The complexity of PFAS exposure patterns in real-world settings necessitates a comprehensive approach to exposure assessment. While many studies have focused on individual PFAS compounds, the importance of considering mixtures is increasingly recognized (13). The National Academies of Sciences, Engineering, and Medicine have emphasized the need for research on the health effects of PFAS mixtures, as opposed to single compounds, to better reflect real-world exposure scenarios (14).

Investigating the crucial biological mechanisms of PFAS on risk of gout is of great importance to the understanding of its toxicity profiles. Uric acid has been reported as a crucial mechanism for health damage caused by environmental exogenous toxicants. Moreover, uric acid was reported as the central aspect of the pathogenesis of gout exposure. Based on the above, uric acid levels may be one biological mechanism of PFAS exposure on gout risk, and the potential intermediary effect of uric acid very likely exists in the relationship of PFAS exposure with gout risk (PFAS to uric acid), however, the role of uric acid levels in the effects of PFAS on gout risk was not fully analyzed, particularly in population-based epidemiological studies. To the best of our knowledge, whether uric acid mediates the adverse effects of PFAS on the risk of gout has not been assessed. Mediation analysis is a causal inference tool in environmental epidemiological studies, as it could divide the total effect into direct effect and indirect effect and be adapted to more complex analysis scenarios with consideration of the potential interaction effects between exposure and mediation.

To address these knowledge gaps, our study aimed to investigate the associations between the concentrations of serum PFASs, both as individual compounds and as mixtures, and the prevalence of gout in a representative sample of the U.S. adult population. By utilizing data from the NHANES from 2007–2018, we employ a robust cross-sectional study design to explore this relationship. Our approach incorporates advanced statistical techniques, including generalized linear models (GLMs) to assessment of PFAS-related gout risk and employ mediation analysis to examine the intermediary effects of uric acid in the relationships of PFAS with gout risk.

This study aims to contribute to the growing body of evidence on the health impacts of PFAS exposure and inform future research and policy decisions regarding these persistent environmental contaminants. By examining both individual PFAS compounds and their mixtures, we aim to provide insights into the complex relationships between environmental exposure and chronic disease risk, with potential implications for public health interventions and regulatory strategies.

## 2 Materials and methods

### 2.1 Study population and design

**Figure 1:**
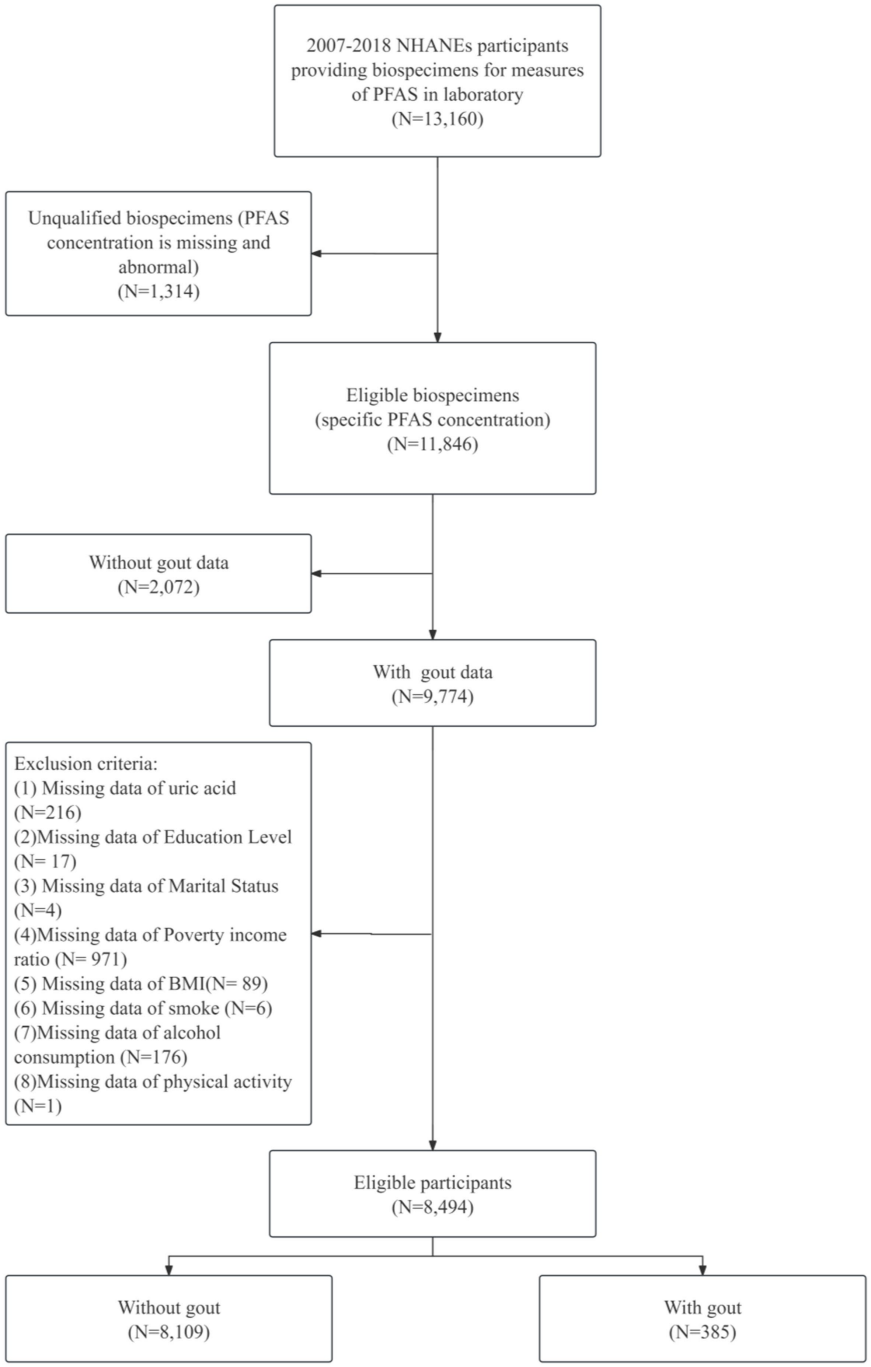
Flowchart of participants selection.

This study utilized data from the NHANES, a cross-sectional, nationally representative survey designed to assess the health and nutritional status of the noninstitutionalized civilian population in the United States. Data from six NHANES cycles spanning 2007–2018 were analyzed. The initial sample comprised 13,160 participants with complete PFASs data across all cycles. A series of exclusion criteria were applied to define the analytical sample. Initially, 1,047 participants lacking biospecimens for PFAS measurements were excluded. By using values beyond 3 times the standard deviation as criteria for determining outliers, 267 samples were excluded. From the remaining 11,846 participants, 2,072 samples lacking gout data were excluded. Additionally, 1,280 individuals with missing data on covariates of interest were removed. This resulted in a final analytical sample of 8,494 participants, consisting of 8,109 individuals without gout and 385 with gout. The detailed sample selection process is shown in Figure 1.

#### 2.1.1 Exposure assessment: serum PFAS concentrations

Serum samples collected from participants were processed and stored at −80°C in specialized containers before being transported to a designated Centers for Disease Control and Prevention (CDC) laboratory for analysis. The specimen collection and processing procedures adhered to the protocols outlined in the NHANES laboratory/medical technician procedure manual.

Quantitative detection of perfluoroalkyl substances, including perfluorooctanoic acid (PFOA), perfluorooctane sulfonic acid (PFOS), perfluorohexane sulfonic acid (PFHxS), and perfluorononanoic acid (PFNA), was performed via online solid-phase extraction coupled with high-performance liquid chromatography-Turbo Ion Spray-Tandem mass Spectrometry (SPE-HPLC-TIS-MS/MS). The limit of detection (LOD) for each PFAS was established at three times the standard deviation (SD) of the blank concentration. For concentrations below the LOD, the machine-read value obtained through instrumental analysis was utilized if detectable. In cases where no machine-read value was available, values were imputed using LOD/ $\sqrt{2}$ .

To account for the right-skewed distribution of PFAS concentrations, all values were log2-transformed for subsequent statistical analyses. Quality control measures, including the analysis of blank samples and duplicate samples, were implemented throughout the analytical process to ensure the accuracy and reliability of PFAS measurements.

Quality control measures were rigorously implemented throughout the analytical process. Each analytical batch included method blanks, duplicate samples, and quality control samples to monitor potential contamination and ensure measurement reliability. Calibration standards were analyzed at the beginning and end of each analytical sequence to verify instrument performance. For internal quality control, relative percent differences for duplicate analyses were maintained below 15%, and recoveries of quality control samples were kept within 85–115% of expected values. All analytical procedures strictly followed the NHANES laboratory protocols to ensure data quality and comparability across different survey cycles.

#### 2.1.2 Outcome assessment: gout

The determination of gout status among participants was based on self-reported data collected through a structured questionnaire administered by trained NHANES interviewers. During the medical conditions interview, participants were asked the following question: “Has a doctor or other health professional ever told you that you had gout?” Participants who responded affirmatively to this question were classified as having gout, whereas those who responded negatively were categorized as not having gout. This binary classification served as the primary outcome variable for subsequent analyses.

#### 2.1.3 Covariate assessment

The selection of covariates was informed by prior research on the associations between PFAS exposure and gout, as well as known risk factors for gout. A comprehensive set of demographic, socioeconomic, lifestyle, and health-related variables was included to adjust for potential confounding effects.

Demographic variables included uric acid, age (analyzed as both a continuous variable and categorized into young: 20–39, middle-aged: 40–59, and older: ≥ 60 years), sex (male, female), and ethnicity (categorized as Mexican American, other Hispanic, non-Hispanic white, non-Hispanic black, and another race). Socioeconomic factors included educational attainment (dichotomized as below high school and high school graduate or higher) and the family poverty-income ratio (PIR, categorized as ≤ 1.30, 1.31–3.50, and > 3.50). Marital status was classified as married, widowed, divorced, separated, never married, or living with a partner. Lifestyle factors included smoking status (Yes: Answer “Yes” to answer “Smoked at least 100 cigarettes in life” or Cotinine value greater than or equal to 0.05 ng/mL; No: Answer “No” to the question or Cotinine value less than 0.05 ng/mL), alcohol status (Yes: Answer the question “How often drink alcohol over past 12 months” with a value greater than or equal to 3; Answer the question “How often drink alcohol over past 12 months” with a value less than 3 are considered nondrinkers), and physical activity (Level 1: Sit during the day and not walk very much; Level 2: Stand or walk a lot during the day but not have to carry or lift things very often; Level 3: Lift light load or have to climb stairs or hills often; Level 4: heavy work or carry heavy loads). The health-related variables included body mass index (BMI), which was analyzed as a continuous variable and categorized as <25, 25–29.9, or ≥ 30 kg/m^2^.

#### 2.1.4 Serum uric acid examination

A standard biochemistry test was conducted by trained laboratory technicians, and uric acid concentration was measured using a timed endpoint method. Detailed instructions about analytical methodologies, principles, and operating procedures are shown in the NHANES Laboratory Method Files.

### 2.2 Statistical methods

The statistical analysis was conducted in two main parts: individual PFAS analysis and mixture analysis. Prior to analysis, all PFAS concentrations were log2-transformed to address their right-skewed distribution. Descriptive statistics were calculated for all study variables, with means and standard deviations reported for continuous variables and frequencies and percentages for categorical variables. Differences in characteristics between participants with and without gout were assessed via *t* tests for continuous variables and chi-square tests for categorical variables.

For the individual PFAS analysis, Pearson correlation coefficients were computed among the log2-transformed PFAS concentrations to evaluate potential collinearity. Associations between individual PFAS concentrations and gout were examined via multivariable logistic regression models. Three models with increasing levels of adjustment were employed: an unadjusted model; a model adjusted for age and sex; and a fully adjusted model including uric acid, age, sex, ethnicity, educational attainment, BMI, PIR, marital status, smoking status, alcohol consumption, and physical activity. Odds ratios (ORs) and 95% confidence intervals (CIs) were calculated to estimate the change in the odds of gout per doubling of PFAS concentration. PFAS concentrations were also modeled categorically using quartiles, with the lowest quartile serving as the reference group.

To account for multiple comparisons, *p* values were adjusted via the Benjamini–Hochberg false discovery rate (FDR) method. Statistical significance was determined at an FDR-adjusted *p* value (*q* value) < 0.05, whereas results with an unadjusted *p* value >0.05 were considered marginally significant. Subgroup analyses were conducted to evaluate potential effect modification by stratifying the fully adjusted model by age group and sex. Restricted cubic spline models with 3–5 knots were used to visualize potential nonlinear relationships between individual PFAS concentrations and the log odds of gout, adjusting for all covariates in the fully adjusted model.

For the mixture analysis, weighted quantile sum (WQS) regression was employed to assess the combined effect of PFAS mixtures on gout risk. Three WQS models were fitted: an unadjusted model, a model adjusted for age and sex, and a fully adjusted model including all covariates as in the individual PFAS analysis. The WQS index was then used in logistic regression models to estimate the mixture effect on gout incidence. The weights assigned to each PFAS in the WQS model were used to assess their relative importance in the mixture effect. These results were compared with which from multiple linear regression by including all PFASs. A mediation analysis was conducted to analyze the effect of uric acid in the relationship of PFAS exposure with gout risk.

Sensitivity analyses were performed for both individual PFAS and mixture analyses. To evaluate the impact of potential outliers, participants with PFAS concentrations above the 99th percentile were excluded. To eliminate the influence of taking the lowest measurable concentration when the PFAS concentration is lower than the measured value, the PFAS concentration below the measured value is randomly reassigned, and all analyses were rerun on the subset to compare with the main analysis results.

Statistical analyses were performed in R (Version 4.4.2). The WQS model was conducted using the “gWQS” package, and the mediation effect model was conducted using the “mediation” package. Statistical significance was regarded as a two-sided *p* value <0.05.

## 3 Results

### 3.1 Statistical description

The study population comprised 8,494 adults, including 4,112 males and 4,382 females. Among these participants, 385 (4.53%) reported a diagnosis of gout. Table 1 presents the demographic and clinical characteristics of the study population stratified by gout status. Significant differences in several key variables were observed between participants with and without gout. Individuals with gout were generally older (63.25 years vs. 48.76 years, p < 0.001) and had a higher BMI (31.93 kg/m^2^ vs. 29.17 kg/m^2^, p < 0.001). The gout group also had a greater proportion of females (51.59% vs. 48.41%, p < 0.001) and showed differences in ethnicity distribution, marital status, physical activity levels, and smoke status compared with the non-gout group.

**Table 1:** Baseline characteristics of participants by gout status.

Variable	Level	Overall	Without gout	With gout	*p* value
Age mean (SE)		49.41 (0.19)	48.76 (0.19)	63.25 (0.69)	< 0.001
Age group, *n* (%)	20–39	2,863 (0.34)	2,836 (0.99)	27 (0.01)	< 0.001
	40–59	2,818 (0.33)	2,711 (0.96)	107 (0.04)	
	≥60	2,813 (0.33)	2,562(0.91)	251 (0.09)	
BMI, mean (SE)		29.36 (0.08)	29.17 (0.08)	31.93 (0.41)	< 0.001
BMI group, *n* (%)	<25	2,441 (0.29)	2,381 ()	60 ()	< 0.001
	25–30	2,746 (0.32)	2,622 ()	124 (0.05)	
	≥30	3,307 (0.39)	3,106(0.93)	201 (0.07)	
PIR, mean (SE)		2.52 (0.02)	2.52 (0.02)	2.59 (0.08)	0.329
PIR Group, *n* (%)	≤1.30	2,727 (0.32)	2,602 (0.95)	125 (0.05)	0.846
	1.31–3.50	3,136 (0.37)	2,999 (0.96)	137 (0.04)	
	>3.50	2,631 (0.31)	2,508 (0.95)	123 (0.05)	
Uric acid mean (SE)		5.46 (0.02)	5.40 (0.02)	6.56 (0.09)	< 0.001
Sex, *n* (%)	Male	4,112(0.48)	3,849(0.94)	263 (0.06)	< 0.001
	Female	4,382(0.52)	4,260 (0.97)	122 (0.03)	
Ethnicity, *n* (%)	Mexican American	1,254 (0.15)	1,231 (0.98)	23(0.02)	< 0.001
	Other Hispanic	869 (0.10)	846 (0.97)	23 (0.03)	
	Non-Hispanic White	3,669 (0.43)	3,458 (0.94)	211(0.06)	
	Non-Hispanic Black	1,720 (0.20)	1,635 (0.95)	85 (0.05)	
	Other Race – Including Multi-Racial	982 (0.11)	939 (0.96)	43 (0.04)	
Marital status, *n* (%)	Married	4,401 (0.52)	4,156 (0.94)	245 (0.06)	< 0.001
	Widowed	643 (0.08)	599 (0.93)	44 (0.07)	
	Divorced	933 (0.12)	889 (0.95)	44 (0.05)	
	Separated	288 (0.03)	274 (0.95)	14 (0.05)	
	Never married	1,561 (0.18)	1,598 (0.98)	28 (0.02)	
	Living with partner	688 (0.08)	658 (0.99)	10 (0.01)	
Smoke status, *n* (%)	Yes	3,757 (0.44)	3,538 (0.94)	219 (0.06)	< 0.001
	No	4,737 (0.56)	4,571 (0.96)	166 (0.04)	
Alcohol status, *n* (%)	Yes	2,924 (0.34)	2,783 (0.95)	141 (0.05)	0.382
	No	5,570 (0.66)	5,326 (0.95)	244 (0.05)	
Physical activity, *n* (%)	Level 1	3,789 (0.45)	3,600 (0.95)	189 (0.05)	0.016
	Level 2	1,914 (0.23)	1,820 (0.95)	94 (0.05)	
	Level 3	1,128 (0.13)	1,096 (0.97)	32 (0.03)	
	Level 4	1,663 (0.20)	1,593 (0.96)	70 (0.04)	
Educational level, *n* (%)	<High school	2012 (0.24)	1,906 (0.95)	106 (0.05)	0.079
	≥High school	6,482 (0.76)	6,203 (0.96)	279 (0.04)	

Age, BMI, PIR and uric acid are continuous variables that are reported as the means and standard deviations. The others are categorical variables, which are reported as frequencies and proportions.

Table 2 summarizes the distributions of the serum concentrations of the four PFAS compounds examined in this study. The table provides key statistics, including geometric means, arithmetic means, and percentiles, offering a comprehensive overview of PFAS exposure levels in the study population. The geometric mean concentration of PFOS was the highest (7.21 ng/mL), followed by that of PFOA (2.24 ng/mL), PFHxS (1.42 ng/mL), and PFNA (0.82 ng/mL) on the log2 scale.

**Table 2:** Distribution of serum per- and polyfluoroalkyl substances (PFASs) among participants.

Log2-PFAS (ng/mL)	GM	Mean (SE)	Percentile			
			25th	50th	75th	95th
PFOA	2.27	0.82 (0.01)	0.35	0.83	1.31	1.95
PFOS	7.40	2.00 (0.01)	1.41	2.03	2.59	3.43
PFHxS	1.41	0.35 (0.01)	−0.22	0.34	0.92	1.74
PFNA	0.85	−0.17 (0.01)	−0.65	−0.11	0.33	1.00

GM, geometric mean.

**Figure 2:**
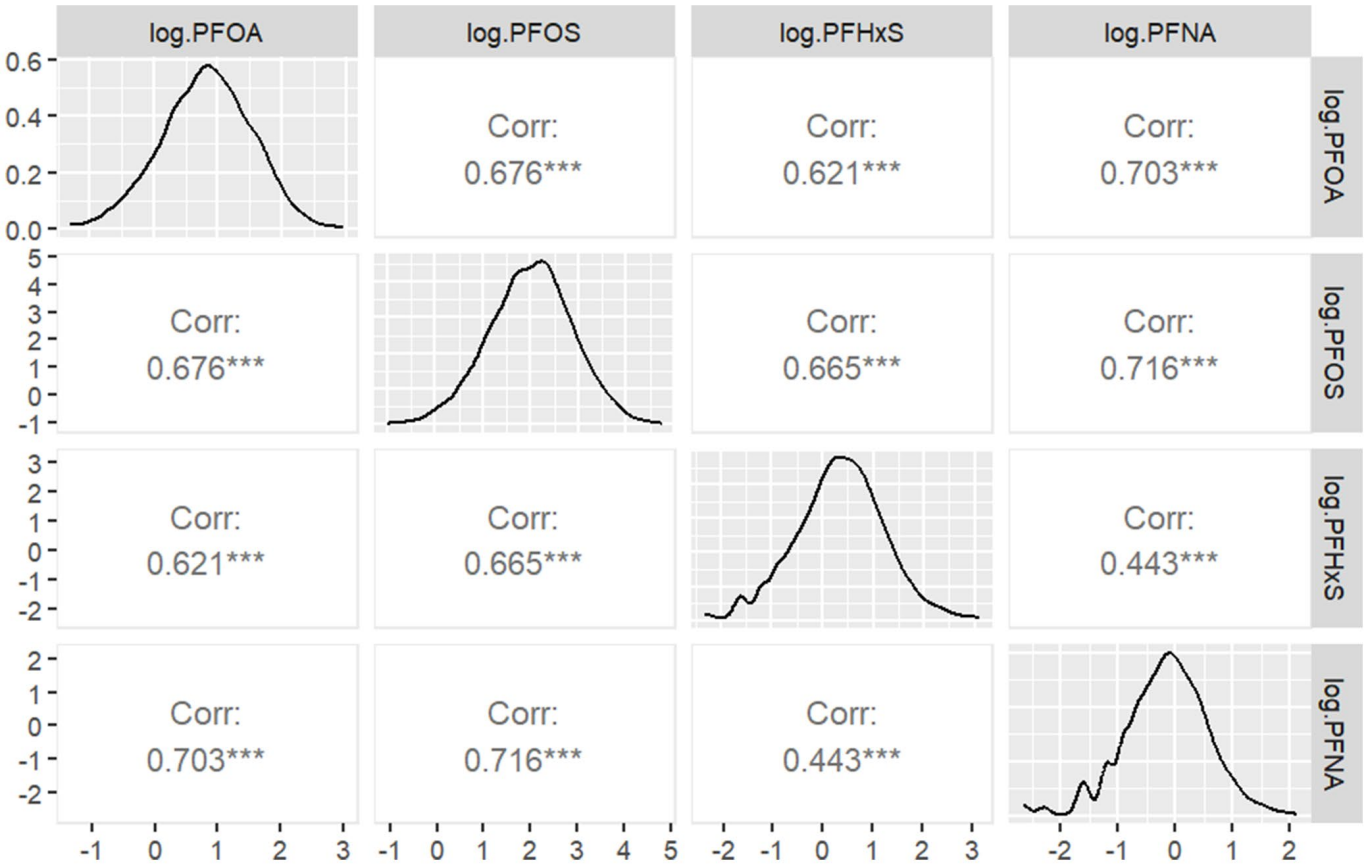
Pearson correlation between serum PFAS levels after Iog2 transformation.

Figure 2 Pearson correlation between serum PFAS levels after Iog2 transformation shows the Pearson correlation coefficients between the log2-transformed serum concentrations of the four PFAS compounds. The correlation analysis revealed varying degrees of association among the PFAS. The strongest correlation was observed between PFOS and PFNA ( r=0.716 ), suggesting potential common sources or similar pharmacokinetics for these compounds. Conversely, PFHxS and PFNA showed the weakest correlation ( r=0.443 ), indicating potentially distinct exposure pathways or metabolic processes for these PFAS.

### 3.2 Individual PFAS analysis

The associations between individual PFAS concentrations and gout were examined via three logistic regression models with increasing levels of adjustment. Table 3 presents the results of these analyses. In the unadjusted model (Model 1), all four PFASs showed significant positive associations with gout. The ORs per doubling of PFAS concentration were 1.50 (95% CI: 1.30, 1.75) for PFOA, 1.47 (95% CI: 1.31, 1.66) for PFOS, 1.33 (95% CI: 1.18, 1.50) for PFHxS, and 1.27 (95% CI: 1.10, 1.46) for PFNA. All associations were statistically significant ( p < 0.001). After adjusting for age and sex (Model 2), the associations were substantially attenuated and lost statistical significance. In the fully adjusted model (Model 3), which accounted for additional demographic, socioeconomic, and lifestyle factors, the associations remained nonsignificant. However, PFOA showed a marginally positive association with gout (OR: 1.11, 95% CI: 0.94, 1.31, p =0.21). The Akaike information criterion (AIC) values indicated improved model fit with increasing adjustment, with Model 3 showing the best fit for all PFAS.

**Table 3:** Results of individual PFAS association analysis via three logistic regressions.

PFAS (ng/mL)		PFOA	PFOS	PFHxS	PFNA
Model 1	OR	1.50	1.47	1.33	1.27
	*p* value	<0.001	<0.001	<0.001	<0.001
	AIC	3,109	3,097	3,116	3,127
Model 2	OR	1.14	0.97	0.91	1.03
	*p* value	0.10	0.69	0.20	0.68
	AIC	2,816	2,820	2,817	2,820
Model 3	OR	1.11	0.95	0.92	0.98
	*p* value	0.21	0.45	0.23	0.75
	AIC	2,644	2,645	2,644	2,644

**Figure 3:**
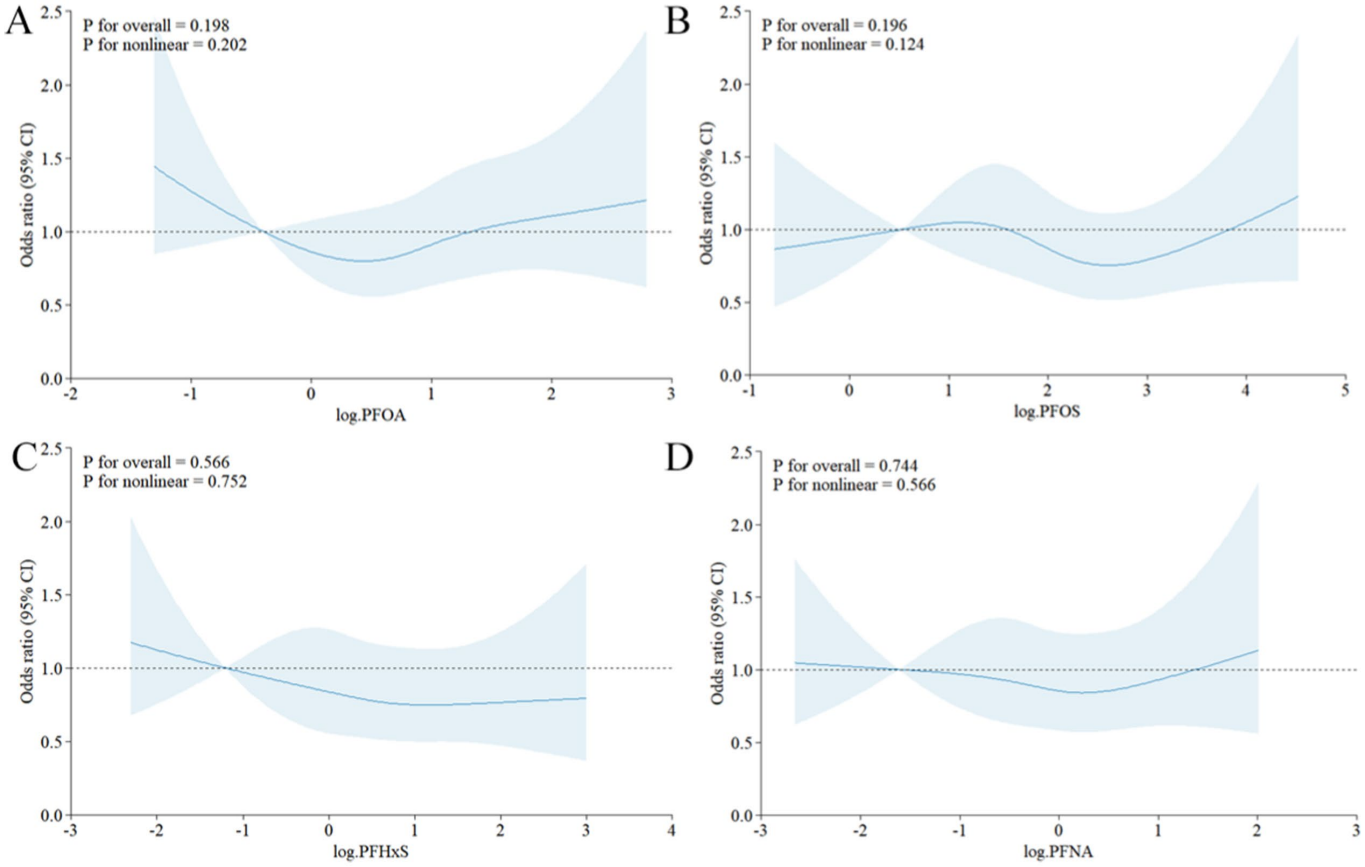
Restricted cubic spline curve of the relationship between serum PFAS levels after Iog2 transformation and the risk of gout. **(A)** PFOA, **(B)** PFOS, **(C)** PFHxS, **(D)** PFNA.

To explore potential nonlinear relationships, restricted cubic spline analyses were performed. Figure 3 illustrates the dose–response relationship between the log2-transformed PFAS concentration and the odds of gout, based on Model 3. The curve suggests no significant nonlinear correlation (PFOA: p for nonlinearity = 0.202, PFOS: p for nonlinearity = 0.124, PFHxS: p for nonlinearity = 0.752, PFNA: p for nonlinearity = 0.566), with a steeper increase in odds at lower PFAS concentrations and a plateauing effect at higher levels.

### 3.3 Mixture PFAS analysis

The combined effects of the four PFASs on gout risk were assessed via WQS regression. Table 4 presents the results of the WQS analysis across the three models with increasing levels of adjustment. In the unadjusted model (Model 1), the WQS index showed a significant positive association with gout (OR: 1.38, 95% CI: 1.28, 1.47). These findings indicate that higher overall PFAS mixture exposure was associated with increased odds of gout. After adjusting for age and sex (Model 2), the association between the PFAS mixture and gout remained significant and became lower (OR:1.35, 95% CI: 1.25, 1.46). In the fully adjusted model (Model 3), which accounted for additional uric acid content, demographic, socioeconomic, and lifestyle factors, the positive association persisted and further strengthened (1.38, 95% CI:1.28, 1.49). The AIC values indicated improved model fit with increasing adjustment, with Model 3 showing the best fit.

**Table 4:** Results of WQS regression.

Model 1			Model 2			Model 3		
OR 95% CI	Std. error	AIC	OR 95% CI	Std. error	AIC	OR 95% CI	Std. error	AIC
1.38 (1.28, 1.47)	0.04	1870	1.35 (1.25, 1.46)	0.04	1863	1.38 (1.28, 1.49)	0.04	1844

**Figure 4:**
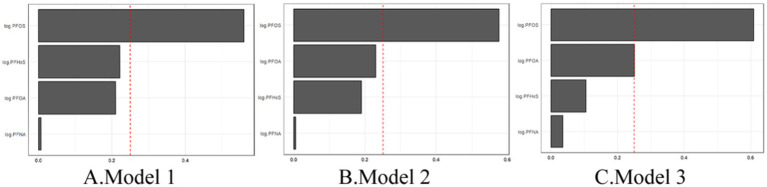
WQS regression showing the magnitude of the assigned weights for each log2-transformed PFAS in relation to gout status for Model 1 **(A)**, Model 2 **(B)**, and Model 3 **(C)**.

Figure 4 illustrates the weights assigned to each PFAS in the WQS index for all three models. Across all the models, PFOS consistently emerged as the largest contributor to the mixture effect. In Model 3, the weights were distributed as follows: PFOS (0.49), PFOA (0.37), PFHxS (0.12), and PFNA (0.02). This suggests that while all four PFAS contributed to the overall mixture effect, PFOS and PFOA play more prominent roles. These findings indicate that combined exposure to multiple PFASs may have a stronger association with gout risk than individual PFAS exposure does. The persistence of this association across different levels of adjustment suggests that the mixture effect is robust to confounding by various demographic and lifestyle factors. The dominance of PFOS in the mixture effect aligns with its higher serum concentrations observed in the study population and underscores its potential importance in gout etiology.

### 3.4 Multiple linear regression analysis

The results from the multiple linear regressions (Table 5) show the multiple linear regression results of three different models (Model 1, Model 2, Model 3), each considering different covariates. Each model analyzed the association between four types of PFAS and gout risk. In Model 1, all four PFAS are positively correlated with gout risk, which is consistent with the WQS regression results. In both Model 2 and Model 3, PFOS, PFHxS, and PFNA were negatively correlated with gout risk, which differs from the WQS regression results. In the multiple linear regression results, the AIC value decreased from Model 1 to Model 3, indicating an improvement in model fit, which is consistent with the WQS regression results.

**Table 5:** Results of individual PFAS association analysis via three multiple linear regressions.

PFAS (ng/mL)		PFOA	PFOS	PFHxS	PFNA
Model 1	OR (95%CI)	1.34 (1.06, 1.69)	1.55 (1.27, 1.90)	0.98 (0.83, 1.16)	0.73 (0.58, 0.92)
	*p* value	0.013	<0.001	0.811	0.007
	AIC	3,093			
Model 2	OR (95%CI)	1.43 (1.13, 1.81)	0.93 (0.75, 1.15)	0.81 (0.68, 0.98)	0.96 (0.76, 1.21)
	*p* value	0.003	0.494	0.026	0.733
	AIC	2,813			
Model 3	OR (95%CI)	1.42 (1.11, 1.81)	0.94 (0.75, 1.17)	0.84 (0.69, 1.01)	0.90 (0.71, 1.15)
	*p* value	0.005	0.552	0.063	0.406
	AIC	2,641			

Overall, WQS regression provides the overall effect of PFAS mixtures, while multiple linear regression provides detailed effects of individual PFAS. These results indicate that higher overall PFAS mixture exposure is associated with an increased risk of gout, even after adjusting for various factors. WQS regression provides a more consistent basis for comparison with multiple linear regression analysis. The combination of the two can provide a more comprehensive analytical perspective.

### 3.5 Subgroup analysis

To explore potential effect modifications, subgroup analyses were conducted, stratifying the fully adjusted model by age and sex. These analyses revealed important variations in the associations between PFASs and gout across different demographic groups.

#### 3.5.1 Individual PFAS analysis

**Figure 5:**
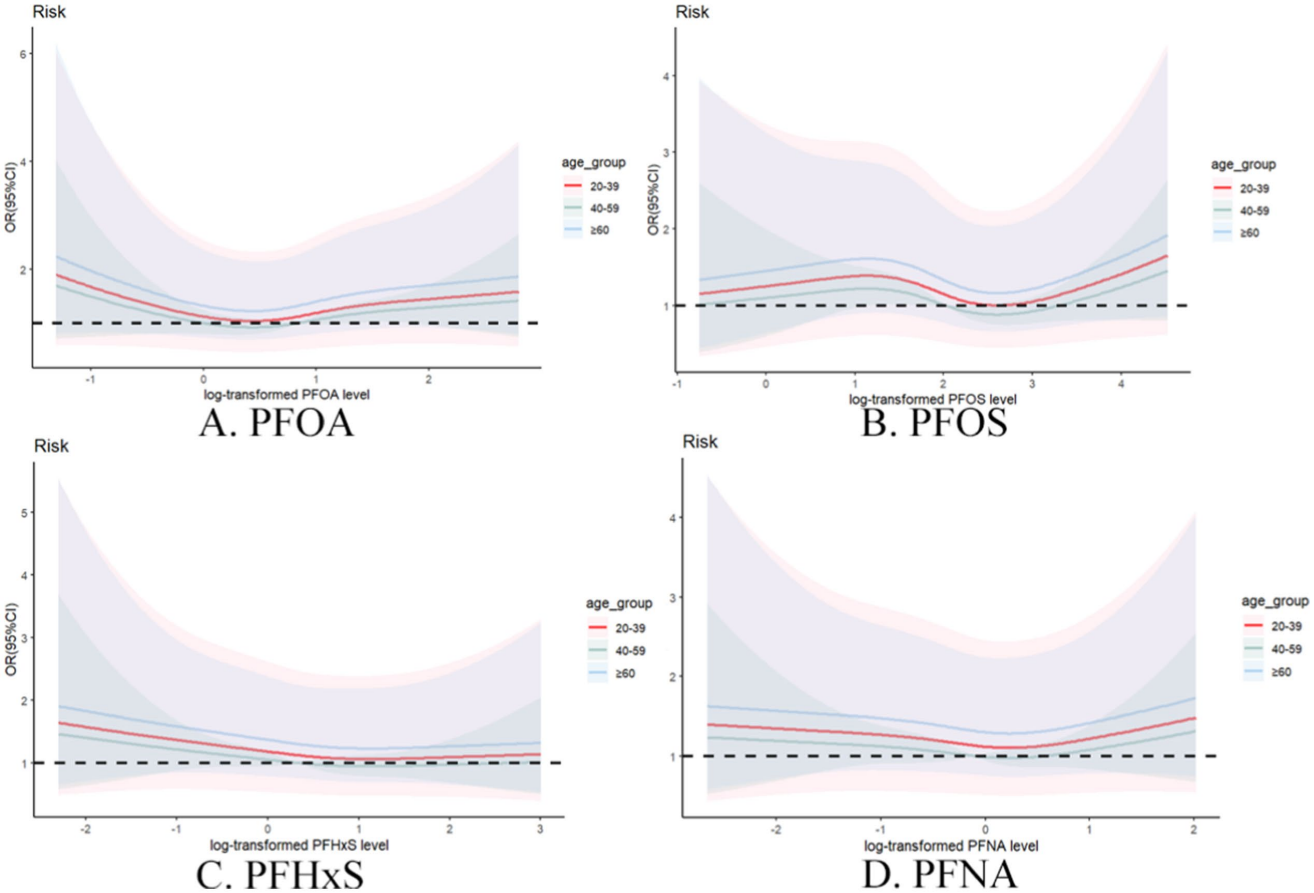
Restrictive cubic spline plot of individual PFASs and the risk of gout in different age groups with full adjustment of covariates. **(A)** PFOA, **(B)** PFOS, **(C)** PFHxS, **(D)** PFNA.

Age-stratified analysis (Figure 5) of individual PFASs revealed distinct patterns across different age groups. For individual PFASs, the association with gout risk was most pronounced in the ≥60 years age group, followed by the 20–39 years age group, with the 40–59 years age group showing the weakest association. This trend suggests that old people may be particularly susceptible to the gout-inducing effect of these PFAS.

**Figure 6:**
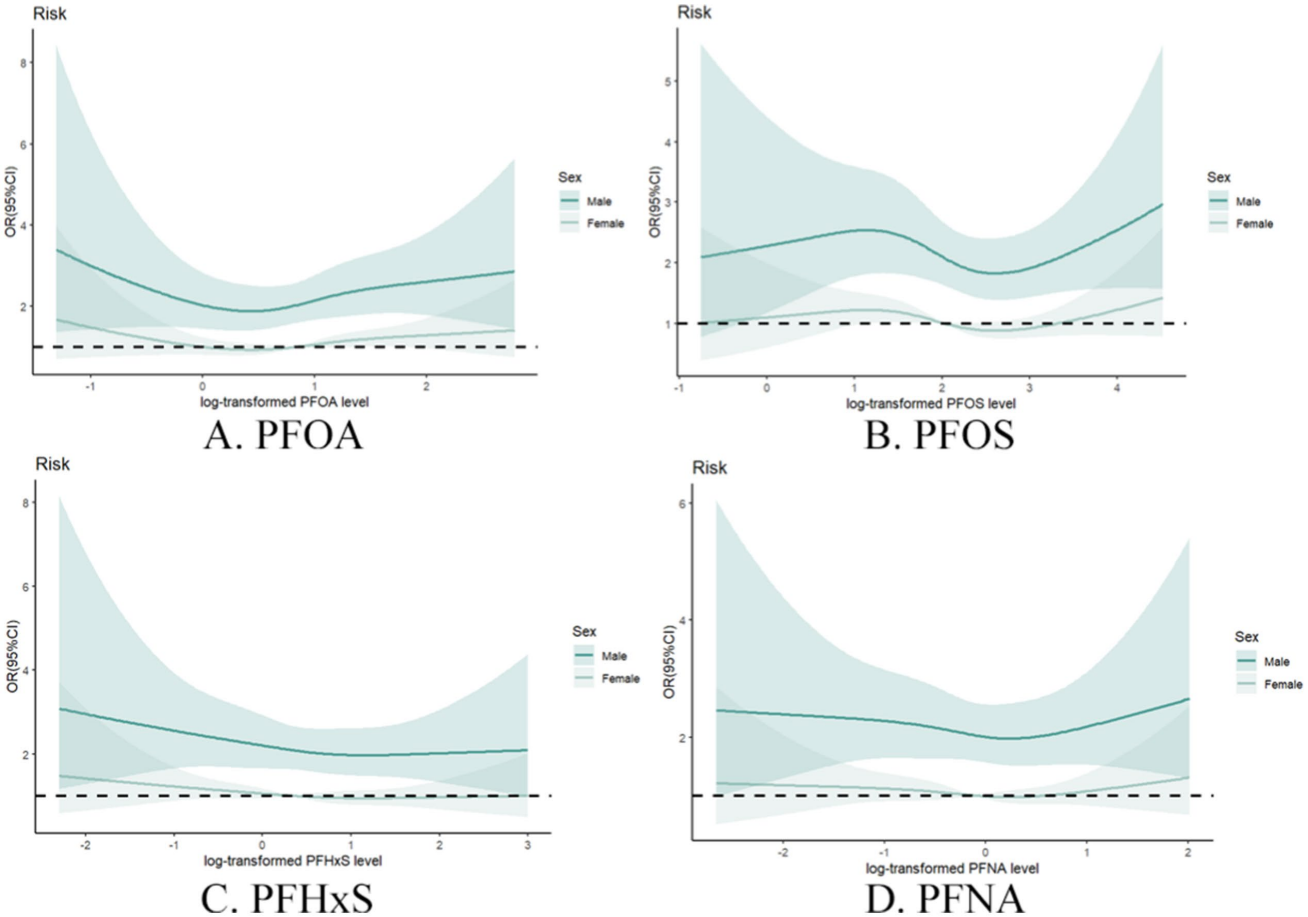
Restrictive cubic spline plot of individual PFASs and the risk of gout in different sex groups with full adjustment of covariates. **(A)** PFOA, **(B)** PFOS, **(C)** PFHxS, **(D)** PFNA.

Sex-stratified analysis (Figure 6) further elucidated the complex relationship between PFAS exposure and gout risk. Compared with females, males generally demonstrated stronger associations between individual PFAS concentrations and gout risk. This sex-specific effect was particularly evident for PFOA and PFOS, suggesting potential interactions between these compounds and metabolism and excretion between males and females.

#### 3.5.2 Mixture PFAS analysis

**Figure 7:**
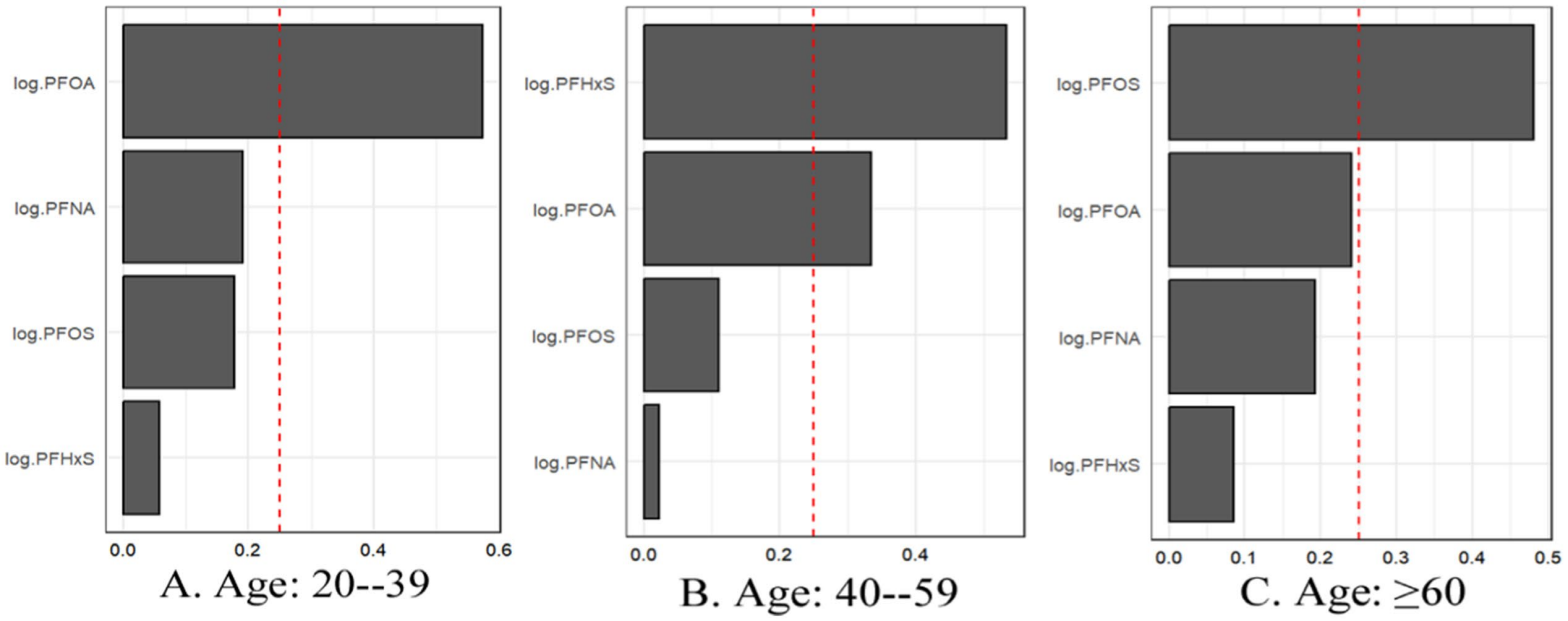
For WQS regression, the magnitude of the assigned weights for each log2-transformed PFAS in relation to the risk of gout for **(A)** Age: 20–39, **(B)** Age: 40–59, and **(C)** Age: ≥60.

The WQS regression analysis provided valuable insights into the combined effects of PFAS mixtures on gout risk across age and sex subgroups (Table 6). In the age-stratified analysis, the association between the PFAS mixture and gout risk was strongest in the 20–39 years age group, followed by the 40–59 years age group, whereas no significant association was observed in the ≥60 years age group (Figure 7). This trend differs from that observed in the individual PFAS analysis, highlighting the importance of considering mixture effects in addition to individual compound effects.

**Table 6:** WQS regression results for different age and sex subgroups.

Variables	Subgroup	OR	95% CI
Age	20–39	1.73	(1.32, 2.28)
	40–59	1.17	(0.98, 1.39)
	≥60	1.06	(0.94, 1.19)
Sex	Male	1.20	(1.05, 1.38)
	Female	1.34	(1.16, 1.55)

**Figure 8:**
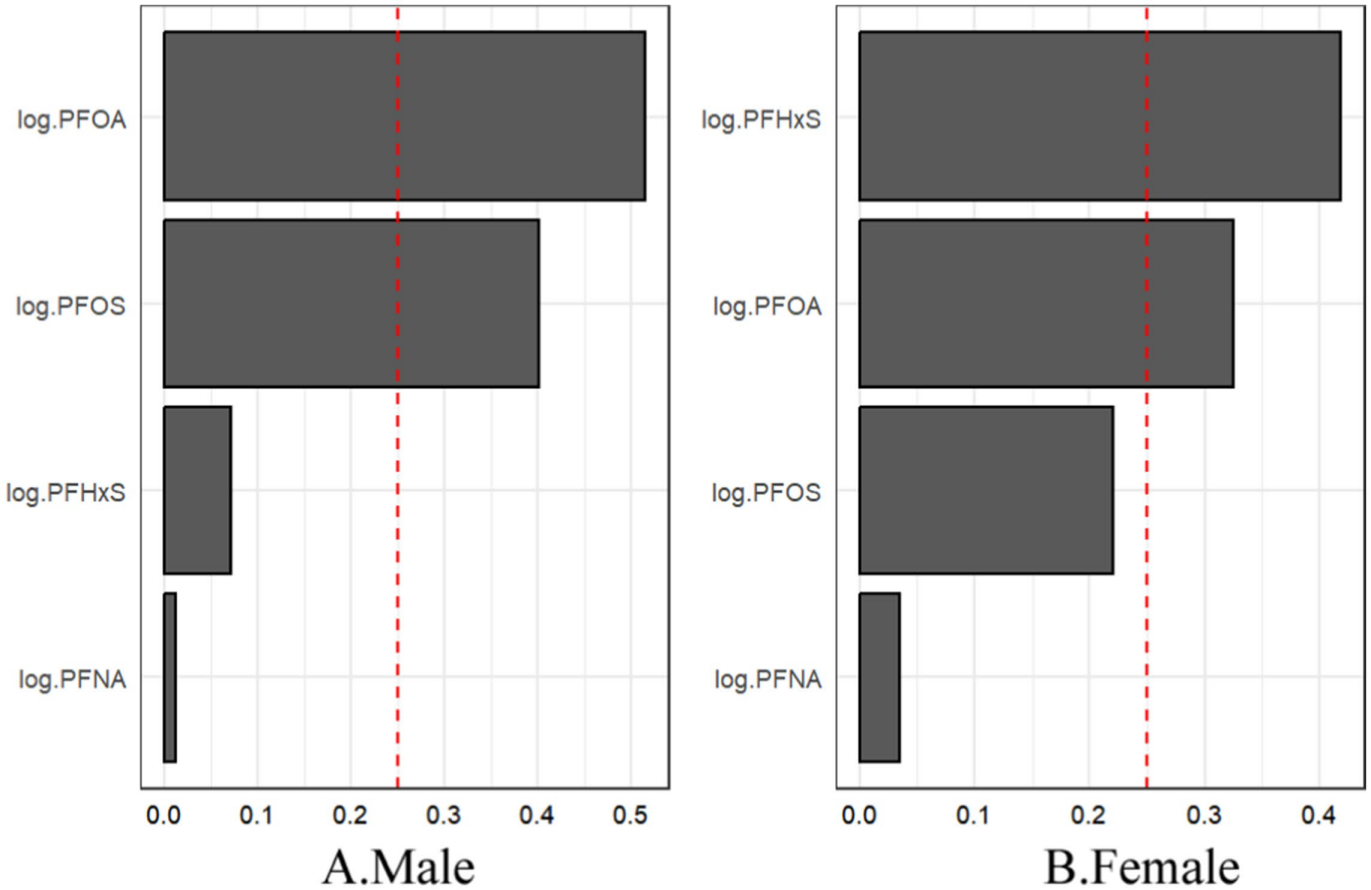
For WQS regression, the magnitude of the assigned weights for each log2-transformed PFAS in relation to the risk of gout for **(A)** males and **(B)** females.

The sex-stratified WQS analysis revealed significant positive associations between the PFAS mixture and gout risk in both males and females (Figure 8). Notably, the relative importance of individual PFASs within the mixture differed between sexes. In males, PFOA contributed the most to the mixture effect, followed by PFOS. In contrast, PFHxS had the highest weight in females, followed by PFOA.

In conclusion, the subgroup analyses revealed a nuanced interplay between age, sex, and PFAS exposure in relation to gout risk. The varying patterns observed in individual and mixture analyses highlight the complexity of PFAS-associated health effects and the need for comprehensive approaches in future studies.

### 3.6 Sensitivity analysis

To evaluate the impact of potential outliers, we conducted sensitivity analyses by excluding participants whose PFAS concentrations were above the 99th percentile (Table 7). For individual PFAS, the patterns of association remained largely consistent with our main analysis, with slight attenuations in the strength of associations, particularly in unadjusted models. The PFAS mixture analysis via WQS regression also revealed robust positive associations with gout risk across all the models, albeit with slightly lower ORs than those in the main analysis. The relative importance of individual PFASs within the mixture remained consistent, with PFOS emerging as the largest contributor to the mixture effect across all the models.

**Table 7:** Results of the sensitivity analysis by excluding participants whose PFAS concentrations were above the 99th percentile.

Analysis	Model	PFOA	PFOS	PFHxS	PFNA
Individual PFAS analysis^a^	Model 1	1.48*	1.42*	1.34*	1.17*
	Model 2	1.12	0.91	0.90	0.95
	Model 3	1.09	0.89	0.90	0.91
Mixture PFAS analysis^b^	Model 1	1.35 (1.23, 1.47)			
	Model 2	1.31 (1.18, 1.45)			
	Model 3	1.36 (1.20, 1.53)			

a Individual PFAS analysis reports ORs; * indicates corresponding *p* values less than 0.05.

b Mixture PFAS analysis reports mixture effect estimates and 95% CIs.

**Figure 9:**
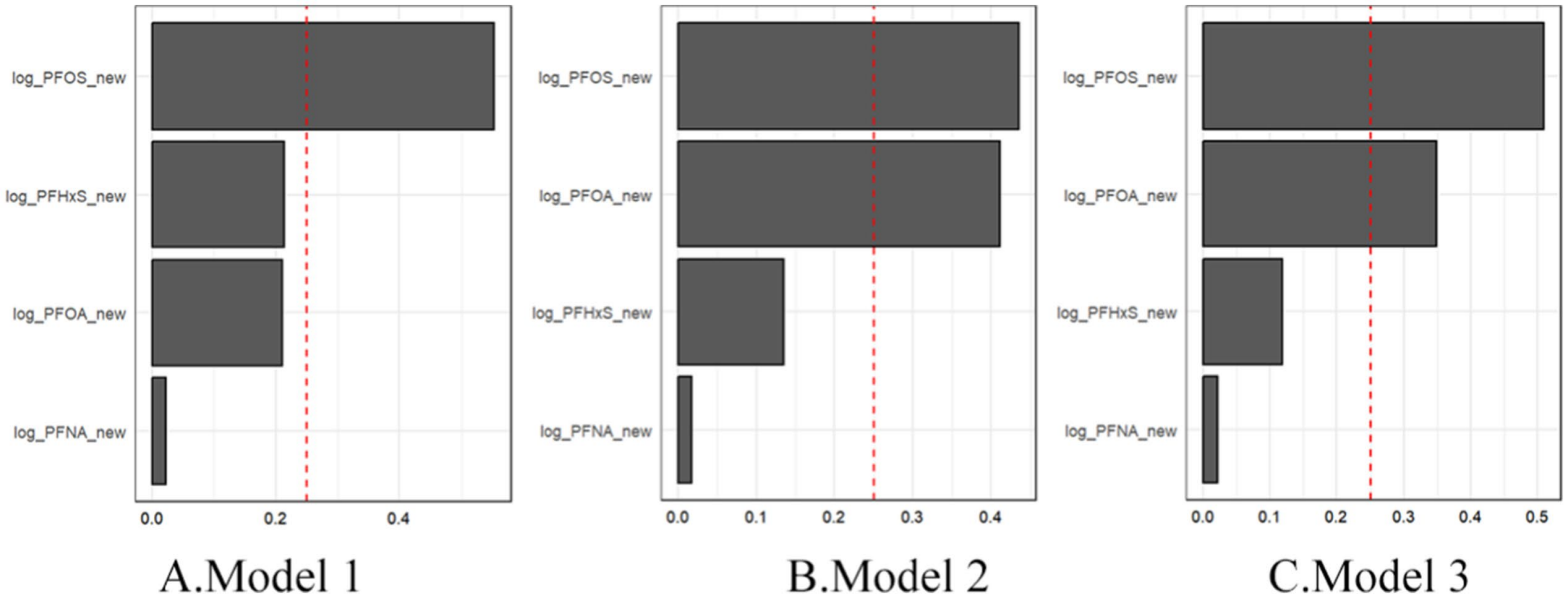
Sensitivity analysis of mixture PFAS showing the magnitude of the assigned weights for each log2-transformed PFAS in relation to gout status for Model 1 **(A)**, Model 2 **(B)**, and Model 3 **(C)**.

To evaluate the impact of PFAS at its lowest measurable concentration, we conducted sensitivity analyses by randomly reassigned PFAS to the lowest measurable concentration (Table 8). In sensitivity analysis, the OR of all PFAS in Model 1 was greater than 1 and the *p*-value was less than 0.05, which is consistent with our main analysis results (Table 3), indicating a significant positive correlation between PFAS concentration and gout risk. All PFAS *p*-values in Model 2 and Model 3 were greater than 0.05, which is consistent with our main analysis results, indicating that the association is no longer significant when controlling for more covariates, but its association has decreased. The relative importance of individual PFASs within the mixture remained consistent, with PFOS emerging as the largest contributor to the mixture effect across all the models (Figure 9).

**Table 8:** Results of the sensitivity analysis by randomly reassigned PFAS to the lowest measurable concentration.

Analysis	Model	PFOA	PFOS	PFHxS	PFNA
Individual PFAS analysis^a^	Model 1	1.50*	1.47*	1.33*	1.27*
	Model 2	1.14	0.97	0.91	1.03
	Model 3	1.11	0.95	0.92	0.98
Mixture PFAS analysis^b^	Model 1	1.37 (1.26, 1.49)			
	Model 2	1.34 (1.25, 1.43)			
	Model 3	1.36(1.25, 1.49)			

a Individual PFAS analysis reports ORs; * indicates corresponding *p* values less than 0.05.

b Mixture PFAS analysis reports mixture effect estimates and 95% CIs.

In summary, these sensitivity analyses indicate that our main findings are generally robust to the influence of extreme PFAS values. While the modest attenuations in the strength of associations, the overall patterns and statistical significance of the results remained largely unchanged for both individual PFAS and PFAS mixtures. This consistency provides additional confidence in the reliability of our primary findings regarding the relationship between PFAS exposure and gout risk.

### 3.7 Analysis of intermediary effect

**Figure 10:**
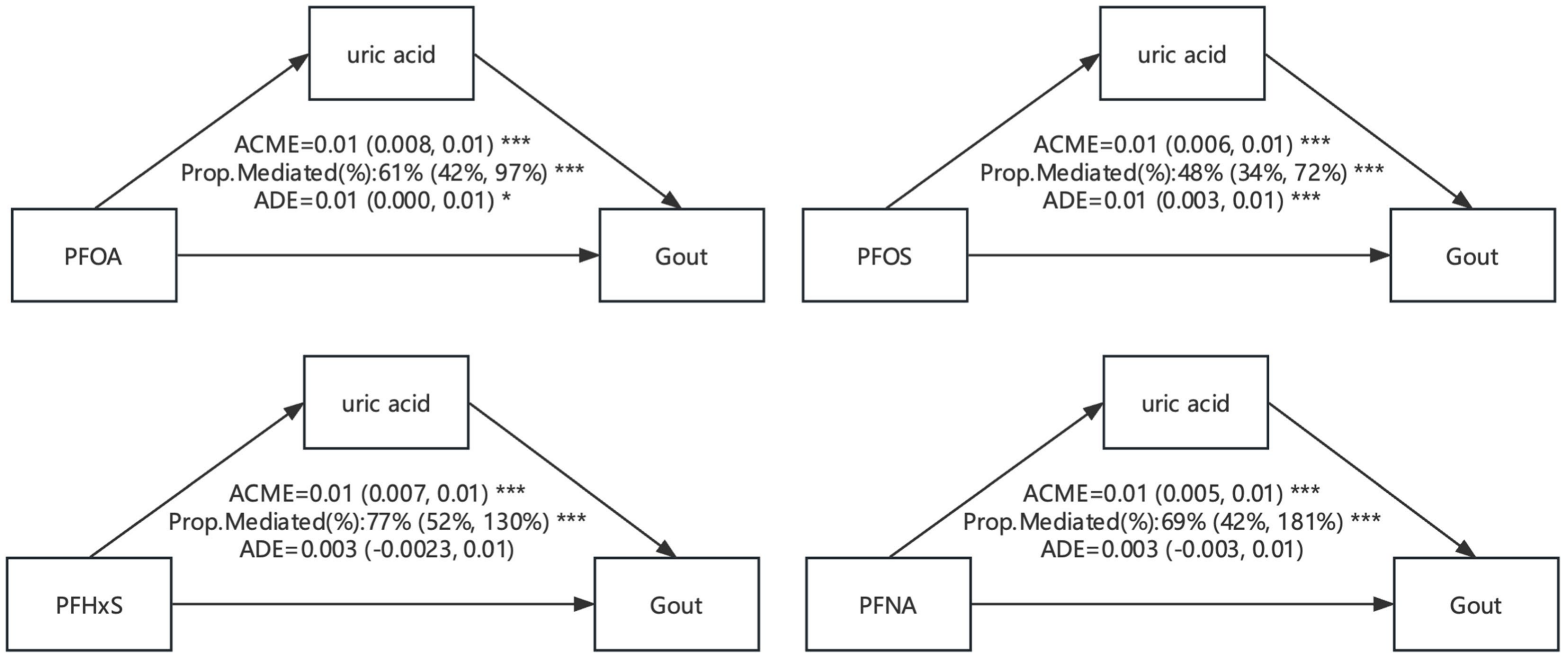
Mediation effects of estimated gout in the associations of PFAS with uric acid level. ACME, average causal mediation effects (indirect effect); ADE, average direct effects. * *p* < 0.05, ** *p* < 0.01, and *** *p* < 0.001.

In this study, we conducted an analysis of intermediary effect of uric acid in illustrating the relationship of PFAS exposure with uric acid and proposed a hypothesis that uric acid might play a significant mediation effect in the associations of PFAS exposure with elevated risk of gout. The causal mediation effect model with consideration of the interaction effect was used in the assessment of the mediation effect of uric acid levels in the associations of PFAS exposure with elevated risk of gout. Significant mediation effects of uric acid were observed in our study for the associations of all 4 PFAS compounds with risk of gout, with the mediated proportion ranging from 48 to 77% (Figure 10).

## 4 Discussion

This study investigated the associations between the serum concentrations of four PFASs and the risk of gout in a representative sample of U.S. adults via data from the NHANES 2007–2018. Our analyses revealed complex relationships between PFAS exposure and gout risk, with important implications for public health and future research directions. According to unadjusted analyses, all four PFASs were significantly positively associated with gout risk. However, after adjusting for demographic and lifestyle factors, these associations were largely attenuated and became nonsignificant, with PFOA showing only a marginally positive association. These findings suggest that the relationships between individual PFAS compounds and gout risk are substantially influenced by various demographic and lifestyle factors. Interestingly, our PFAS mixture analysis via WQS regression revealed a significant positive association between overall PFAS exposure and gout risk, which highlights the importance of considering combined PFAS exposure, as the cumulative effect may be more relevant to health outcomes than individual compound exposure. Our results align with recent research emphasizing the need to study PFAS mixtures, such as the work of Grandjean et al. (15), which demonstrated synergistic effects of PFAS mixtures on liver injury and cholesterol levels.

Subgroup analyses indicated potential effect modification by age and sex, with stronger associations observed in old peoples and females. These findings suggest potential age- and sex-specific vulnerabilities to PFAS-induced gout risk, which could be related to differences in PFAS pharmacokinetics, hormonal influences, or lifestyle factors across these subgroups. The biological plausibility of our findings is supported by previous research linking PFAS exposure to elevated uric acid levels and increased risk of hyperuricemia (16, 17). PFASs may interfere with uric acid metabolism and excretion through various mechanisms, including the inhibition of organic anion transporters in the kidneys (18), the activation of nuclear receptors such as PPAR α (3), and the induction of oxidative stress and inflammation (19). Notably, PFOS consistently emerged as the largest contributor to the mixture effect across the different models. This prominence is consistent with its higher serum concentrations and longer half-life than those of other PFAS compounds (20), underscoring the potential long-term health impacts of legacy PFASs despite regulatory efforts to phase out their use.

Several limitations should be considered when interpreting our results. The cross-sectional nature of the study limits our ability to establish causal or temporal relationships between PFAS exposure and gout development. Reliance on self-reported gout diagnoses may introduce misclassification bias, and future studies could benefit from incorporating clinical diagnoses or serum uric acid measurements. Our analysis was limited to four PFAS compounds, and expanding the panel to include emerging PFASs and other environmental contaminants could provide a more comprehensive understanding of exposure effects (6, 21). Despite adjusting for numerous covariates, the potential for residual confounding remains. The absence of longitudinal data limits our ability to assess cumulative PFAS exposure and its long-term effects on gout risk. Finally, while the NHANES provides a nationally representative sample, our findings may not be generalizable to populations with different exposure profiles or genetic backgrounds.

Despite these limitations, our study makes several important contributions to the field. This study provides one of the first comprehensive assessments of both individual and mixed PFAS effects on gout risk in a large, representative U.S. population. The use of advanced statistical techniques, including WQS regression, offers insights into the cumulative impact of PFAS mixtures, addressing a critical gap in the literature. Our subgroup analyses highlight potential susceptible populations, informing future targeted research and public health interventions. Moreover, this study underscores the importance of considering both individual compounds and mixtures when evaluating PFAS-related health risks (22).

Future research directions could include longitudinal studies to establish temporal relationships and assess cumulative PFAS exposure effects on gout risk. Mechanistic studies are needed to elucidate the biological pathways linking PFAS exposure to uric acid dysregulation and gout development (23). Investigating potential gene–environment interactions that may modify PFAS-related gout risk could provide valuable insights into susceptibility factors. Evaluating the impact of PFAS exposure reduction strategies on gout incidence and prevalence is crucial for informing public health interventions. Finally, expanding the PFAS panel to include emerging compounds and exploring potential synergistic effects with other environmental contaminants would contribute to a more comprehensive understanding of PFAS-related health risks (24).

In conclusion, our study provides evidence for a potential link between PFAS exposure and gout risk, particularly when considering cumulative exposure to PFAS mixtures. These findings contribute to the growing body of evidence on PFAS-related health effects and highlight the need for continued research and regulatory efforts to mitigate PFAS exposure in the general population. As we continue to unravel the complex relationships between environmental exposure and chronic diseases, studies such as ours play crucial roles in informing public health policies and guiding future research endeavors in environmental health.

## Data Availability

The original contributions presented in the study are included in the article/Supplementary material, further inquiries can be directed to the corresponding authors.
