# Supplementary material for: Association of per- and polyfluoroalkyl substances with gout risk: a cross-sectional analysis of NHANES 2007–2018 data emphasizing mixture effects
Source: Front Public Health. 2025 Feb 10;13:1484663. doi: 10.3389/fpubh.2025.1484663 (PMC11847820; doi:10.3389/fpubh.2025.1484663)
Supplement: Supplementary file 2 [file Table_1.docx]

#自变量：PFAS："log.PFOA", "log.PFOS", "log.PFHS", "log.PFNA"

#因变量：MCQ160N

#协变量：LBXSUA尿酸,RIAGENDR性别，RIDAGEYR年龄，BMI，RIDRETH1种族，DMDEDUC2教育程度，DMDMARTL婚姻状况，INDFMPIR年收入贫困线比数

#2. Materials and Methods

#2.1 Study population and design##########################################################################################################

#gout只有07-18年的数据，故以下全部统一用07-18年的数据

library(haven)

library(dplyr)

#(1)录入PFAS数据##############################################################################################

setwd("C:\\Users\\Administrator\\Desktop\\PFAS-Gout文章修改")

PFAS <- read.csv("PFAS_07_18.csv")

#(2)录入MCQ160数据###############################################################################

setwd("C:\\Users\\Administrator\\Desktop\\gout-尿酸-PFAS\\MCQ_03_18")

MCQ3<- read_xpt("MCQ_E.XPT") %>%

dplyr::select(SEQN,MCQ160N)

MCQ4<- read_xpt("MCQ_F.XPT") %>%

dplyr::select(SEQN,MCQ160N)

MCQ5<- read_xpt("MCQ_G.XPT") %>%

dplyr::select(SEQN,MCQ160N)

MCQ6<- read_xpt("MCQ_H.XPT") %>%

dplyr::select(SEQN,MCQ160N)

MCQ7<- read_xpt("MCQ_I.XPT") %>%

dplyr::select(SEQN,MCQ160N)

MCQ8<- read_xpt("MCQ_J.XPT") %>%

dplyr::select(SEQN,MCQ160N)

MCQ <- rbind(MCQ3,MCQ4,MCQ5,MCQ6,MCQ7,MCQ8)

#(3)录入协变量数据#############################################################################

#1*尿酸数据导入##########################################################################33

setwd("C:\\Users\\Administrator\\Desktop\\gout-尿酸-PFAS\\LBXSUA")

UA3<- read_xpt("BIOPRO_E.xpt") %>%

dplyr::select(SEQN,LBXSUA)

UA4<- read_xpt("BIOPRO_F.xpt") %>%

dplyr::select(SEQN,LBXSUA)

UA5<- read_xpt("BIOPRO_G.xpt") %>%

dplyr::select(SEQN,LBXSUA)

UA6<- read_xpt("BIOPRO_H.xpt") %>%

dplyr::select(SEQN,LBXSUA)

UA7<- read_xpt("BIOPRO_I.xpt") %>%

dplyr::select(SEQN,LBXSUA)

UA8<- read_xpt("BIOPRO_J.xpt") %>%

dplyr::select(SEQN,LBXSUA)

UA <- rbind(UA3,UA4,UA5,UA6,UA7,UA8)

#2*录入RIAGENDR性别，RIDAGEYR年龄，RIDRETH1种族，DMDEDUC2教育程度，DMDMARTL婚姻状况，INDFMPIR年收入贫困线比数数据

setwd("C:\\Users\\Administrator\\Desktop\\gout-尿酸-PFAS\\DEMO_03_18")

E2<- read_xpt("DEMO_E.XPT") %>%

dplyr::select(SEQN, RIAGENDR,RIDAGEYR,RIDRETH1,DMDEDUC2,DMDMARTL,INDFMPIR)

F2<- read_xpt("DEMO_F.XPT") %>%

dplyr::select(SEQN, RIAGENDR,RIDAGEYR,RIDRETH1,DMDEDUC2,DMDMARTL,INDFMPIR)

G2<- read_xpt("DEMO_G.XPT") %>%

dplyr::select(SEQN, RIAGENDR,RIDAGEYR,RIDRETH1,DMDEDUC2,DMDMARTL,INDFMPIR)

H2<- read_xpt("DEMO_H.XPT") %>%

dplyr::select(SEQN, RIAGENDR,RIDAGEYR,RIDRETH1,DMDEDUC2,DMDMARTL,INDFMPIR)

I2<- read_xpt("DEMO_I.XPT") %>%

dplyr::select(SEQN, RIAGENDR,RIDAGEYR,RIDRETH1,DMDEDUC2,DMDMARTL,INDFMPIR)

J2<- read_xpt("DEMO_J.XPT") %>%

dplyr::select(SEQN, RIAGENDR,RIDAGEYR,RIDRETH1,DMDEDUC2,DMDMARTL,INDFMPIR)

DEMO <- rbind(E2,F2,G2,H2,I2,J2)

#3*录入BMI数据############################################################################################################

setwd("C:\\Users\\Administrator\\Desktop\\gout-尿酸-PFAS\\BMX_03_18")

BMX3<- read_xpt("BMX_E.xpt") %>%

dplyr::select(SEQN,BMXBMI)

BMX4<- read_xpt("BMX_F.xpt") %>%

dplyr::select(SEQN,BMXBMI)

BMX5<- read_xpt("BMX_G.xpt") %>%

dplyr::select(SEQN,BMXBMI)

BMX6<- read_xpt("BMX_H.xpt") %>%

dplyr::select(SEQN,BMXBMI)

BMX7<- read_xpt("BMX_I.xpt") %>%

dplyr::select(SEQN,BMXBMI)

BMX8<- read_xpt("BMX_J.xpt") %>%

dplyr::select(SEQN,BMXBMI)

BMI <- rbind(BMX3,BMX4,BMX5,BMX6,BMX7,BMX8)

#4.录入吸烟数据############################################################################################################

#SMQ020___1:Y,2:F,7：拒绝,9：不知道，.:失踪

setwd("C:\\Users\\Administrator\\Desktop\\PFAS-Gout文章修改\\PFAS与骨质酥松关联第五阶段分析结果\\SMQ")

E1<- read_xpt("SMQ_E.XPT") %>%

dplyr::select(SEQN, SMQ020)

F1<- read_xpt("SMQ_F.XPT") %>%

dplyr::select(SEQN, SMQ020)

G1<- read_xpt("SMQ_G.XPT") %>%

dplyr::select(SEQN, SMQ020)

H1<- read_xpt("SMQ_H.XPT") %>%

dplyr::select(SEQN, SMQ020)

I1<- read_xpt("SMQ_I.XPT") %>%

dplyr::select(SEQN, SMQ020)

J1<- read_xpt("SMQ_J.XPT") %>%

dplyr::select(SEQN, SMQ020)

SMQ <- rbind(E1,F1,G1,H1,I1,J1)

#血清中的可替宁 （ng/mL）（大于0.05微克/升-为吸烟）#1:大于0.05#2：小于0.05#3：为空值

setwd("C:\\Users\\Administrator\\Desktop\\PFAS-Gout文章修改\\PFAS与骨质酥松关联第五阶段分析结果\\COTNAL")

COT3<- read_xpt("COTNAL_E.XPT") %>%

dplyr::select(SEQN, LBXCOT)

COT4<- read_xpt("COTNAL_F.XPT") %>%

dplyr::select(SEQN, LBXCOT)

COT5<- read_xpt("COTNAL_G.XPT") %>%

dplyr::select(SEQN,LBXCOT)

COT6 <- read_xpt("COT_H.XPT") %>%

dplyr::select(SEQN,LBXCOT)

COT7<- read_xpt("COT_I.XPT") %>%

dplyr::select(SEQN, LBXCOT )

COT8<- read_xpt("COT_J.XPT") %>%

dplyr::select(SEQN, LBXCOT )

COT <- rbind(COT3,COT4,COT5,COT6,COT7,COT8)

#5*录入饮酒数据

#把喝酒次数（天数）大于等于3的定义为1：有喝过酒，其余为2：没喝过酒

setwd("C:\\Users\\Administrator\\Desktop\\PFAS-Gout文章修改\\PFAS与骨质酥松关联第五阶段分析结果\\ALQ")

ALQ_E<- read_xpt("ALQ_E.XPT") %>%

dplyr::select(SEQN,ALQ120Q)

ALQ_F<- read_xpt("ALQ_F.XPT") %>%

dplyr::select(SEQN,ALQ120Q)

ALQ_G<- read_xpt("ALQ_G.XPT") %>%

dplyr::select(SEQN,ALQ120Q)

ALQ_H<- read_xpt("ALQ_H.XPT") %>%

dplyr::select(SEQN,ALQ120Q)

ALQ_I<- read_xpt("ALQ_I.XPT") %>%

dplyr::select(SEQN,ALQ120Q)

ALQ <- rbind(ALQ_E,ALQ_F,ALQ_G,ALQ_H,ALQ_I)

ALQ <- ALQ %>%

mutate(ALQ120Q= case_when(

ALQ120Q >= 3 & ALQ120Q < 776 ~ 1,

TRUE ~ 2

))

ALQ_J <- read_xpt("ALQ_J.XPT") %>%

dplyr::select(SEQN,ALQ121)

ALQ_J <- ALQ_J %>%

mutate(ALQ121 = case_when(

ALQ121 %in% c(10, 0) ~ 2,

ALQ121 %in% c(1, 2, 3, 4, 5, 6, 7, 8, 9) ~ 1,

TRUE ~ ALQ121

))

ALQ_J <- rename(ALQ_J, ALQ120Q=ALQ121)

ALQ <- rbind(ALQ,ALQ_J)

#6*导入运动数据

setwd("C:\\Users\\Administrator\\Desktop\\gout-尿酸-PFAS\\PAQ_03_18")

PAQ3<- read_xpt("PAQ_E.xpt") %>%

dplyr::select(SEQN,PAQ605,PAQ620,PAQ635)

PAQ4<- read_xpt("PAQ_F.xpt") %>%

dplyr::select(SEQN,PAQ605,PAQ620,PAQ635)

PAQ5<- read_xpt("PAQ_G.xpt") %>%

dplyr::select(SEQN,PAQ605,PAQ620,PAQ635)

PAQ6<- read_xpt("PAQ_H.xpt") %>%

dplyr::select(SEQN,PAQ605,PAQ620,PAQ635)

PAQ7<- read_xpt("PAQ_I.xpt") %>%

dplyr::select(SEQN,PAQ605,PAQ620,PAQ635)

PAQ8<- read_xpt("PAQ_J.xpt") %>%

dplyr::select(SEQN,PAQ605,PAQ620,PAQ635)

PAQ <- rbind(PAQ3,PAQ4,PAQ5,PAQ6,PAQ7,PAQ8)

PAQ <- PAQ %>%

mutate(PAQ180 = NA)

PAQ <- PAQ %>%

mutate(PAQ180 = case_when(

PAQ605 == 1 ~ 4, # PAQ605为1的赋值为4

PAQ620 == 1 & is.na(PAQ180) ~ 2, # 除已赋值的行外，PAQ620为1的赋值为2

PAQ635 == 1 & is.na(PAQ180) ~ 3, # 除已赋值的行外，PAQ635为1的赋值为3

(PAQ605 == 2 & PAQ620 == 2 & PAQ635 == 2) & is.na(PAQ180) ~ 1,

# PAQ605，PAQ620和PAQ635列同时为2且未赋值的赋值为1

TRUE ~ PAQ180 # 保留之前赋的NA值

))

PAQ <- PAQ[, c("SEQN", "PAQ180")]

####2.合并数据#####################################################################################################

merge1 <- left_join(PFAS,MCQ, by = "SEQN")

merge2 <- left_join(merge1,UA, by = "SEQN")

merge3 <- left_join(merge2,DEMO, by = "SEQN")

merge4 <- left_join(merge3,BMI, by = "SEQN")

merge5 <- left_join(merge4,SMQ, by = "SEQN")

merge6 <- left_join(merge5,COT, by = "SEQN")

merge7 <- left_join(merge6,ALQ, by = "SEQN")

merge <- left_join(merge7,PAQ , by = "SEQN")

nrow(merge)#[1] 13160

write.csv(merge, "C:\\Users\\Administrator\\Desktop\\PFAS-Gout文章修改\\merge.csv")

####3.清洁数据######################################################################################

#(1)pfas:剔除log.PFOA,log.PFOS,log.PFHS，log.PFNA列为NA的行

cleaned_merge <- merge %>%

filter(!is.na(log.PFOA) & !is.na(log.PFOS) & !is.na(log.PFHxS) & !is.na(log.PFNA))

# 计算指定列的均值和标准差

means <- sapply(cleaned_merge[c("log.PFOA", "log.PFOS", "log.PFHxS", "log.PFNA")], mean, na.rm = TRUE)

sds <- sapply(cleaned_merge[c("log.PFOA", "log.PFOS", "log.PFHxS", "log.PFNA")], sd, na.rm = TRUE)

# 创建一个逻辑向量，判断每行是否包含异常值

is_outlier <- function(x, mean, sd) {

abs(x - mean) > 3 * sd

}

outlier_rows <- apply(cleaned_merge[c("log.PFOA", "log.PFOS", "log.PFHxS", "log.PFNA")], 1, function(row) {

any(sapply(1:length(row), function(i) is_outlier(row[i], means[i], sds[i])))

})

# 剔除包含异常值的行,以3倍标准差之外的值作为异常值的判断标准

cleaned_merge <- cleaned_merge[!outlier_rows, ]

nrow(cleaned_merge)#[1] 11846

#(2)痛风数据预处理

cleaned_merge <- cleaned_merge %>%

filter(!is.na(MCQ160N) )

cleaned_merge <- cleaned_merge %>%

dplyr::filter(MCQ160N != 9,MCQ160N != 7)

nrow(cleaned_merge)#[1] 9774

#(3)UA

cleaned_merge <- cleaned_merge %>%

filter(!is.na(LBXSUA) )

nrow(cleaned_merge)#[1] 9758

#(4)DEMO

#RIDAGEYR年龄

cleaned_merge <- cleaned_merge %>%

filter(!is.na(RIDAGEYR) )

nrow(cleaned_merge)#[1] 9758

#RIAGENDR

cleaned_merge <- cleaned_merge %>%

filter(!is.na(RIAGENDR) )

nrow(cleaned_merge)#[1] 9758

#RIDRETH1

cleaned_merge <- cleaned_merge %>%

filter(!is.na(RIDRETH1) )

nrow(cleaned_merge)#[1] 9758

#DMDEDUC2

cleaned_merge <- cleaned_merge %>%

filter(!is.na(DMDEDUC2) )

cleaned_merge <- cleaned_merge %>%

dplyr::filter(DMDEDUC2!= 9,DMDEDUC2!= 7)

nrow(cleaned_merge)#[1] 9741

#DMDMARTL

cleaned_merge <- cleaned_merge %>%

filter(!is.na(DMDMARTL) )

cleaned_merge <- cleaned_merge %>%

dplyr::filter(DMDMARTL!= 99,DMDMARTL!= 77)

nrow(cleaned_merge)#[1]9737

#INDFMPIR

cleaned_merge <- cleaned_merge %>%

filter(!is.na(INDFMPIR) )

nrow(cleaned_merge)#[1] 8766

#(5)BMI

cleaned_merge <- cleaned_merge %>%

filter(!is.na(BMXBMI) )

nrow(cleaned_merge)#[1] 8677

#(6)SMQ

cleaned_merge <- cleaned_merge %>%

filter(!is.na(SMQ020) )

cleaned_merge <- cleaned_merge %>%

dplyr::filter(SMQ020!= 9,SMQ020!= 7)

nrow(cleaned_merge)#[1] 8673

#(7)COT

cleaned_merge <- cleaned_merge %>%

filter(!is.na(LBXCOT) )

cleaned_merge <- cleaned_merge %>%

mutate(LBXCOT = case_when(

LBXCOT >= 0.05 ~ 1,

LBXCOT < 0.05 ~ 2,

))#（大于0.05微克/升-为吸烟）#1:大于0.05#2：小于0.05

nrow(cleaned_merge)#[1] 8671

#定义吸烟######################

cleaned_merge <- cleaned_merge %>%

mutate(SMOKE = case_when(

SMQ020 == 1 | LBXCOT == 1 ~ 1,

TRUE ~ 2

))

#(8)ALQ

cleaned_merge <- cleaned_merge %>%

filter(!is.na(ALQ120Q) )

cleaned_merge <- cleaned_merge %>%

dplyr::filter(ALQ120Q != 999,ALQ120Q != 777,ALQ120Q != 77,ALQ120Q != 99)

nrow(cleaned_merge)#[1] 8495

#(9)PAQ

cleaned_merge <- cleaned_merge %>%

filter(!is.na(PAQ180) )

nrow(cleaned_merge)#[1] 8494

# 检查每一列是否存在空值

na_check <- cleaned_merge %>%

summarise(across(everything(), ~any(is.na(.))))

print(na_check)

# 计算MCQ160N列中值为1和2的个数

sum(cleaned_merge$MCQ160N == 1)#[1] 385-Yes

sum(cleaned_merge$MCQ160N == 2)#[1] 8109-no

##3.1 Statistical description###########################################################################3

library(dplyr)

library(stats)

# 创建年龄分组变量

cleaned_merge <- cleaned_merge %>%

mutate(age_group = case_when(

RIDAGEYR >= 20 & RIDAGEYR < 40 ~ "20-39",

RIDAGEYR >= 40 & RIDAGEYR < 60 ~ "40-59",

RIDAGEYR >= 60 ~ "≥60"

))

# 创建 BMI 分组变量

cleaned_merge <- cleaned_merge %>%

mutate(bmi_group = case_when(

BMXBMI >= 25 & BMXBMI < 30 ~ "25-30.0",

BMXBMI < 25 ~ "<25",

BMXBMI >= 30 ~ "≥30.0"

))

# 创建教育程度分组变量

cleaned_merge <- cleaned_merge %>%

mutate(edu_group = case_when(

DMDEDUC2 < 3 ~ "<3",

DMDEDUC2 >= 3 ~ "≥3"

))

# 创建收入贫困比例分组变量

cleaned_merge <- cleaned_merge %>%

mutate(pir_group = case_when(

INDFMPIR <= 1.30 ~ "≤1.30",

INDFMPIR > 1.30 & INDFMPIR <= 3.50 ~ "1.31–3.50",

INDFMPIR > 3.50 ~ "> 3.50"

))

write.csv(cleaned_merge, "C:\\Users\\Administrator\\Desktop\\PFAS-Gout文章修改\\cleaned_merge.csv")

sum(cleaned_merge$RIAGENDR == 1)#[1] 4112-Male

sum(cleaned_merge$RIAGENDR == 2)#[1] 4382-Female

mean(cleaned_merge$RIDAGEYR)#49.413

sd(cleaned_merge$RIDAGEYR) / sqrt(length(cleaned_merge$RIDAGEYR))#[1] 0.1912174

cleaned_merge %>%

group_by(MCQ160N) %>%

summarise(

Mean_Age = sprintf("%.2f", mean(RIDAGEYR, na.rm = TRUE)),

SE_Age = sprintf("%.2f", sd(RIDAGEYR, na.rm = TRUE) / sqrt(sum(!is.na(RIDAGEYR))))

)

sum(cleaned_merge$age_group == "20-39")

sum(cleaned_merge$age_group == "20-39")/nrow(cleaned_merge)

sum(cleaned_merge$age_group == "40-59")

sum(cleaned_merge$age_group == "40-59")/nrow(cleaned_merge)

sum(cleaned_merge$age_group == "≥60")

sum(cleaned_merge$age_group == "≥60")/nrow(cleaned_merge)

cleaned_merge %>%

group_by(MCQ160N, age_group) %>%

summarise(

Frequency = n(),

)

#检验cleaned_merge数据框中的RIAGENDR列是否服从正态分布

ks.test(scale(cleaned_merge$RIAGENDR), "pnorm")#D = 0.34953, p-value < 2.2e-16

#不符合正态分布用Wilcoxon秩和检验，符合正态分布用 Student’s t test ,

wilcox.test(cleaned_merge %>%

filter(MCQ160N == 1) %>%

pull(RIAGENDR), cleaned_merge %>%

filter(MCQ160N == 2) %>%

pull(RIAGENDR))#W = 1235582, p-value = 1.28e-15

#卡方检验用于评估各组分类变量的差异

chisq.test(table(cleaned_merge$age_group, cleaned_merge$MCQ160N))#X-squared = 214.07, df = 2, p-value < 2.2e-16

mean(cleaned_merge$BMXBMI)

sd(cleaned_merge$BMXBMI) / sqrt(length(cleaned_merge$BMXBMI))

cleaned_merge %>%

group_by(MCQ160N) %>%

summarise(

Mean_Age = sprintf("%.2f", mean(BMXBMI, na.rm = TRUE)),

SE_Age = sprintf("%.2f", sd(BMXBMI, na.rm = TRUE) / sqrt(sum(!is.na(BMXBMI))))

)

ks.test(scale(cleaned_merge$BMXBMI), "pnorm")

wilcox.test(cleaned_merge %>%

filter(MCQ160N == 1) %>%

pull(BMXBMI), cleaned_merge %>%

filter(MCQ160N == 2) %>%

pull(BMXBMI))

sum(cleaned_merge$bmi_group == "<25")

sum(cleaned_merge$bmi_group == "<25")/nrow(cleaned_merge)

sum(cleaned_merge$bmi_group == "25-30.0")

sum(cleaned_merge$bmi_group == "25-30.0")/nrow(cleaned_merge)

sum(cleaned_merge$bmi_group == "≥30.0")

sum(cleaned_merge$bmi_group == "≥30.0")/nrow(cleaned_merge)

cleaned_merge %>%

group_by(MCQ160N, pir_group) %>%

summarise(

Frequency = n(),

)

chisq.test(table(cleaned_merge$pir_group, cleaned_merge$MCQ160N))

mean(cleaned_merge$INDFMPIR)

sd(cleaned_merge$INDFMPIR) / sqrt(length(cleaned_merge$INDFMPIR))

cleaned_merge %>%

group_by(MCQ160N) %>%

summarise(

Mean_Age = sprintf("%.2f", mean(INDFMPIR, na.rm = TRUE)),

SE_Age = sprintf("%.2f", sd(INDFMPIR, na.rm = TRUE) / sqrt(sum(!is.na(INDFMPIR))))

)

ks.test(scale(cleaned_merge$INDFMPIR), "pnorm")

wilcox.test(cleaned_merge %>%

filter(MCQ160N == 1) %>%

pull(INDFMPIR), cleaned_merge %>%

filter(MCQ160N == 2) %>%

pull(INDFMPIR))

sum(cleaned_merge$pir_group == "≤1.30")

sum(cleaned_merge$pir_group == "≤1.30")/nrow(cleaned_merge)

sum(cleaned_merge$pir_group == "1.31–3.50")

sum(cleaned_merge$pir_group == "1.31–3.50")/nrow(cleaned_merge)

sum(cleaned_merge$pir_group == "> 3.50")

sum(cleaned_merge$pir_group == "> 3.50")/nrow(cleaned_merge)

cleaned_merge %>%

group_by(MCQ160N, pir_group) %>%

summarise(

Frequency = n(),

)

chisq.test(table(cleaned_merge$pir_group, cleaned_merge$MCQ160N))

sum(cleaned_merge$RIAGENDR == "1")#Male

sum(cleaned_merge$RIAGENDR == "1")/nrow(cleaned_merge)

sum(cleaned_merge$RIAGENDR == "2")#Female

sum(cleaned_merge$RIAGENDR == "2")/nrow(cleaned_merge)

cleaned_merge %>%

group_by(MCQ160N, RIAGENDR) %>%

summarise(

Frequency = n(),

)

chisq.test(table(cleaned_merge$RIAGENDR, cleaned_merge$MCQ160N))

sum(cleaned_merge$RIDRETH1 == "1")

sum(cleaned_merge$RIDRETH1 == "1")/nrow(cleaned_merge)

sum(cleaned_merge$RIDRETH1 == "2")

sum(cleaned_merge$RIDRETH1 == "2")/nrow(cleaned_merge)

sum(cleaned_merge$RIDRETH1 == "3")

sum(cleaned_merge$RIDRETH1 == "3")/nrow(cleaned_merge)

sum(cleaned_merge$RIDRETH1 == "4")

sum(cleaned_merge$RIDRETH1 == "4")/nrow(cleaned_merge)

sum(cleaned_merge$RIDRETH1 == "5")

sum(cleaned_merge$RIDRETH1 == "5")/nrow(cleaned_merge)

cleaned_merge %>%

group_by(MCQ160N, RIDRETH1) %>%

summarise(

Frequency = n(),

)

chisq.test(table(cleaned_merge$RIDRETH1, cleaned_merge$MCQ160N))

sum(cleaned_merge$DMDMARTL == "1")

sum(cleaned_merge$DMDMARTL == "1")/nrow(cleaned_merge)

sum(cleaned_merge$DMDMARTL == "2")

sum(cleaned_merge$DMDMARTL == "2")/nrow(cleaned_merge)

sum(cleaned_merge$DMDMARTL == "3")

sum(cleaned_merge$DMDMARTL == "3")/nrow(cleaned_merge)

sum(cleaned_merge$DMDMARTL == "4")

sum(cleaned_merge$DMDMARTL == "4")/nrow(cleaned_merge)

sum(cleaned_merge$DMDMARTL == "5")

sum(cleaned_merge$DMDMARTL == "5")/nrow(cleaned_merge)

sum(cleaned_merge$DMDMARTL == "6")

sum(cleaned_merge$DMDMARTL == "6")/nrow(cleaned_merge)

cleaned_merge %>%

group_by(MCQ160N, DMDMARTL) %>%

summarise(

Frequency = n(),

)

chisq.test(table(cleaned_merge$DMDMARTL, cleaned_merge$MCQ160N))

sum(cleaned_merge$SMQ020 == "1")#yes

sum(cleaned_merge$SMQ020 == "1")/nrow(cleaned_merge)

sum(cleaned_merge$SMQ020 == "2")

sum(cleaned_merge$SMQ020 == "2")/nrow(cleaned_merge)

cleaned_merge %>%

group_by(MCQ160N,SMQ020) %>%

summarise(

Frequency = n(),

)

chisq.test(table(cleaned_merge$SMQ020, cleaned_merge$MCQ160N))

sum(cleaned_merge$ALQ120Q== "1")#yes

sum(cleaned_merge$ALQ120Q== "1")/nrow(cleaned_merge)

sum(cleaned_merge$ALQ120Q== "2")

sum(cleaned_merge$ALQ120Q== "2")/nrow(cleaned_merge)

cleaned_merge %>%

group_by(MCQ160N,ALQ120Q) %>%

summarise(

Frequency = n(),

)

chisq.test(table(cleaned_merge$ALQ120Q, cleaned_merge$MCQ160N))

sum(cleaned_merge$PAQ180== "1")

sum(cleaned_merge$PAQ180== "1")/nrow(cleaned_merge)

sum(cleaned_merge$PAQ180== "2")

sum(cleaned_merge$PAQ180== "2")/nrow(cleaned_merge)

sum(cleaned_merge$PAQ180== "3")

sum(cleaned_merge$PAQ180== "3")/nrow(cleaned_merge)

sum(cleaned_merge$PAQ180== "4")

sum(cleaned_merge$PAQ180== "4")/nrow(cleaned_merge)

cleaned_merge %>%

group_by(MCQ160N,PAQ180) %>%

summarise(

Frequency = n(),

)

chisq.test(table(cleaned_merge$PAQ180, cleaned_merge$MCQ160N))

sum(cleaned_merge$edu_group== "<3")

sum(cleaned_merge$edu_group== "<3")/nrow(cleaned_merge)

sum(cleaned_merge$edu_group== "≥3")

sum(cleaned_merge$edu_group== "≥3")/nrow(cleaned_merge)

cleaned_merge %>%

group_by(MCQ160N,edu_group) %>%

summarise(

Frequency = n(),

)

chisq.test(table(cleaned_merge$edu_group, cleaned_merge$MCQ160N))

mean(cleaned_merge$LBXSUA)

sd(cleaned_merge$LBXSUA) / sqrt(length(cleaned_merge$LBXSUA))

cleaned_merge %>%

group_by(MCQ160N) %>%

summarise(

Mean_Age = sprintf("%.2f", mean(LBXSUA, na.rm = TRUE)),

SE_Age = sprintf("%.2f", sd(LBXSUA, na.rm = TRUE) / sqrt(sum(!is.na(BMXBMI))))

)

ks.test(scale(cleaned_merge$LBXSUA), "pnorm")

wilcox.test(cleaned_merge %>%

filter(MCQ160N == 1) %>%

pull(LBXSUA), cleaned_merge %>%

filter(MCQ160N == 2) %>%

pull(LBXSUA))

#参与者血清中全氟和多氟烷基物质（PFASs）的分布

library(dplyr)

library(broom)

exp(mean(cleaned_merge$log.PFOA, na.rm = TRUE)) # 几何平均数

exp(mean(cleaned_merge$log.PFOS, na.rm = TRUE))

exp(mean(cleaned_merge$log.PFHxS, na.rm = TRUE))

exp(mean(cleaned_merge$log.PFNA, na.rm = TRUE))

mean(cleaned_merge$log.PFOA, na.rm = TRUE) # 算术平均数

mean(cleaned_merge$log.PFOS, na.rm = TRUE)

mean(cleaned_merge$log.PFHxS, na.rm = TRUE)

mean(cleaned_merge$log.PFNA, na.rm = TRUE)

sd(cleaned_merge$log.PFOA, na.rm = TRUE) / sqrt(sum(!is.na(cleaned_merge$log.PFOA))) # 标准误差

sd(cleaned_merge$log.PFOS, na.rm = TRUE) / sqrt(sum(!is.na(cleaned_merge$log.PFOS)))

sd(cleaned_merge$log.PFHxS, na.rm = TRUE) / sqrt(sum(!is.na(cleaned_merge$log.PFHxS)))

sd(cleaned_merge$log.PFNA, na.rm = TRUE) / sqrt(sum(!is.na(cleaned_merge$log.PFNA)))

quantile(cleaned_merge$log.PFOA, probs = c(0.25, 0.5, 0.75, 0.95), na.rm = TRUE) # 百分位数

quantile(cleaned_merge$log.PFOS, probs = c(0.25, 0.5, 0.75, 0.95), na.rm = TRUE) # 百分位数

quantile(cleaned_merge$log.PFHxS, probs = c(0.25, 0.5, 0.75, 0.95), na.rm = TRUE) # 百分位数

quantile(cleaned_merge$log.PFNA, probs = c(0.25, 0.5, 0.75, 0.95), na.rm = TRUE) # 百分位数

####5.相关性分析############################################################################3

library(ggplot2)

library(GGally)

ggpairs(cleaned_merge, columns = c("log.PFOA", "log.PFOS", "log.PFHxS", "log.PFNA"),

lower = list(continuous = "cor"),

upper = list(continuous = "cor"))

#3.2. Individual PFAS analysis#######################################

##6.individual PFAS association analysis via three logistic regressions####################################

library(gWQS)

library(ggplot2)

library(knitr)

library(kableExtra)

library(reshape2)

library(broom)

library(MASS)

cleaned_merge<-cleaned_merge%>%

mutate(MCQ160N= abs(MCQ160N-2))

calculate_stats <- function(formula, data) {

model <- glm(formula, data = data, family = "binomial")

summary_model <- summary(model)

or_values <- exp(coef(model))

p_values <- summary_model$coefficients[, 4]

aic_value <- AIC(model)

conf_intervals <- exp(confint(model))

cat("Model:", deparse(formula), "\n")

cat("AIC:", round(aic_value, 3), "\n")

cat("Coefficients:\n")

print(round(coef(summary_model), 3))

cat("Odds Ratios:\n")

print(round(or_values, 3))

cat("95% Confidence Intervals:\n")

print(round(conf_intervals, 3))

cat("\n")

}

#Model1: 不控制协变量

calculate_stats(MCQ160N ~ log.PFOA, cleaned_merge)

calculate_stats(MCQ160N ~ log.PFOS, cleaned_merge) # PFOS

calculate_stats(MCQ160N ~ log.PFHxS, cleaned_merge) # PFHS

calculate_stats(MCQ160N ~ log.PFNA, cleaned_merge) # PFNA

#Model2: 控制年龄和性别

cleaned_merge$RIAGENDR <- as.factor(cleaned_merge$RIAGENDR)

calculate_stats(MCQ160N~ log.PFOA + RIDAGEYR + RIAGENDR, data =cleaned_merge)

calculate_stats(MCQ160N~ log.PFOS + RIDAGEYR + RIAGENDR, data =cleaned_merge)

calculate_stats(MCQ160N~ log.PFHxS + RIDAGEYR + RIAGENDR, data =cleaned_merge)

calculate_stats(MCQ160N~ log.PFNA + RIDAGEYR + RIAGENDR, data =cleaned_merge)

#Model3: 控制上述全部协变量

calculate_stats(MCQ160N ~ log.PFOA + LBXSUA+RIDAGEYR + RIAGENDR+BMXBMI+RIDRETH1+DMDEDUC2+DMDMARTL+INDFMPIR, data =cleaned_merge)

calculate_stats(MCQ160N ~ log.PFOS + LBXSUA+RIDAGEYR + RIAGENDR+BMXBMI+RIDRETH1+DMDEDUC2+DMDMARTL+INDFMPIR, data =cleaned_merge)

calculate_stats(MCQ160N ~ log.PFHxS + LBXSUA+RIDAGEYR + RIAGENDR+BMXBMI+RIDRETH1+DMDEDUC2+DMDMARTL+INDFMPIR, data =cleaned_merge)

calculate_stats(MCQ160N ~ log.PFNA + LBXSUA+RIDAGEYR + RIAGENDR+BMXBMI+RIDRETH1+DMDEDUC2+DMDMARTL+INDFMPIR, data =cleaned_merge)

## 6.绘制线性回归模型的 RCS 曲线#############################################################33

library(plotRCS)

rcsplot(data = cleaned_merge,

outcome = "MCQ160N",

exposure = "log.PFOA",

covariates = c("LBXSUA","RIDAGEYR","RIAGENDR","BMXBMI","RIDRETH1","DMDEDUC2","DMDMARTL","INDFMPIR"))

rcsplot(data = cleaned_merge,

outcome = "MCQ160N",

exposure = "log.PFOS",

covariates = c("LBXSUA","RIDAGEYR","RIAGENDR","BMXBMI","RIDRETH1","DMDEDUC2","DMDMARTL","INDFMPIR"))

rcsplot(data = cleaned_merge,

outcome = "MCQ160N",

exposure = "log.PFHxS",

covariates = c("LBXSUA","RIDAGEYR","RIAGENDR","BMXBMI","RIDRETH1","DMDEDUC2","DMDMARTL","INDFMPIR"))

rcsplot(data = cleaned_merge,

outcome = "MCQ160N",

exposure = "log.PFNA",

covariates = c("LBXSUA","RIDAGEYR","RIAGENDR","BMXBMI","RIDRETH1","DMDEDUC2","DMDMARTL","INDFMPIR"))

#3.3 Mixture PFAS analysis############################################33

##7.Mixture PFAS analysis########################################3

library(gWQS)

library(ggplot2)

library(knitr)

library(kableExtra)

library(reshape2)

CEP <-c("log.PFOA","log.PFOS","log.PFHxS","log.PFNA")

cleaned_merge$ALQ120Q<-factor(cleaned_merge$ALQ120Q)

cleaned_merge$SMQ020<-factor(cleaned_merge$SMQ020)

cleaned_merge$PAQ180<-factor(cleaned_merge$PAQ180)

results_model1 <- gwqs(MCQ160N ~ wqs+ALQ120Q+SMQ020+PAQ180, mix_name = CEP, data =cleaned_merge,q = 4,

validation = 0.6, b = 10, b1_pos = TRUE, rh = 10, family = "binomial")

results_model2 <- gwqs(MCQ160N ~ wqs + ALQ120Q + SMQ020 + PAQ180, mix_name = CEP, data =cleaned_merge, q = 4,

validation = 0.6, b = 10, b1_pos = TRUE, rh = 10, family = "binomial",

covariables = ~ RIDAGEYR + RIAGEND)

results_model3 <- gwqs(MCQ160N ~ wqs + ALQ120Q + SMQ020 + PAQ180, mix_name = CEP, data =cleaned_merge, q = 4,

validation = 0.6, b = 10, b1_pos = TRUE, rh = 10, family = "binomial",

covariables = ~ LBXSUA+RIDAGEYR + RIAGENDR + RIDRETH1 + BMXBMI + DMDEDUC2 + DMDMARTL + INDFMPIR)

summary(results_model1)

summary(results_model2)

summary(results_model3)

gwqs_barplot(results_model1)

gwqs_barplot(results_model2)

gwqs_barplot(results_model3)

#3.4 multiple linear regression analysis #######################################################################################

# 加载必要的库

library(dplyr)

library(broom)

# 定义数据框 cleaned_merge

# 假设 cleaned_merge 已经包含所需的变量

# 定义模型

model1 <- glm(MCQ160N ~ log.PFOA + log.PFOS + log.PFHxS + log.PFNA, family = "binomial", data = cleaned_merge)

model2 <- glm(MCQ160N ~ log.PFOA + log.PFOS + log.PFHxS + log.PFNA + RIAGENDR + RIDAGEYR, data = cleaned_merge, family = "binomial")

model3 <- glm(MCQ160N ~ log.PFOA + log.PFOS + log.PFHxS + log.PFNA + LBXSUA + RIAGENDR + RIDAGEYR + BMXBMI + RIDRETH1 + DMDEDUC2 + DMDMARTL + INDFMPIR, data = cleaned_merge, family = "binomial")

# 定义计算统计量的函数

calculate_stats <- function(model) {

summary_model <- summary(model)

coefficients <- round(summary_model$coefficients[, 1:4], 3)

or_values <- round(exp(coefficients[, "Estimate"]), 3)

aic_value <- round(AIC(model), 3)

ci_values <- confint(model, level = 0.95) # 计算 95% 置信区间

ci_values <- round(exp(ci_values), 3) # 转换为 OR 的置信区间

list(

Estimates = coefficients[, "Estimate"],

StdErrors = coefficients[, "Std. Error"],

ZValues = coefficients[, "z value"],

PValues = coefficients[, "Pr(>|z|)"],

OddsRatios = or_values,

CI_Lower = ci_values[, 1],

CI_Upper = ci_values[, 2],

AIC = aic_value

)

}

# 计算每个模型的统计量

results_model1 <- calculate_stats(model1)

results_model2 <- calculate_stats(model2)

results_model3 <- calculate_stats(model3)

# 打印结果

print("Model 1 Results:")

print(results_model1)

print("\nModel 2 Results:")

print(results_model2)

print("\nModel 3 Results:")

print(results_model3)

#3.5 Subgroup analysis############################################################################################################

#3.5.1 Individual PFAS analysis####################################

library(MASS)

library(rms)

library(ggplot2)

library(gridExtra)

cleaned_merge$ age_group <- as.factor(cleaned_merge$ age_group)

dd <- datadist(cleaned_merge)

options(datadist = "dd")

#PFOA

model <-lrm(MCQ160N~rcs(log.PFOA,4)+age_group+LBXSUA+RIDAGEYR + RIAGENDR + RIDRETH1 + BMXBMI + DMDEDUC2 + DMDMARTL + INDFMPIR,data= cleaned_merge)

summary(model)

AGE <- Predict(model,

log.PFOA,

age_group = levels(cleaned_merge$ age_group),

fun = exp,

type = "predictions",

ref.zero = TRUE,

conf.int = 0.95)

summary(AGE)

#使用 gplot 绘制图形

library(ggplot2)

ggplot() +

geom_line(data = AGE, aes(log.PFOA, yhat, color = age_group),

linetype = "solid", size = 1, alpha = 0.9) +

geom_ribbon(data = AGE,

aes(log.PFOA, ymin = lower, ymax = upper, fill = age_group),

alpha = 0.2) +

scale_color_manual(values = c("red", "#9Ec4be","#abd0f1", "#dce9f4"),

labels = c("20-39", "40-59","≥60")) +

scale_fill_manual(values = c("pink", "#9Ec4be", "#abd0f1", "#dce9f4"),

labels = c("20-39", "40-59","≥60")) +

theme_classic() +

geom_hline(yintercept = 1, linetype = 2, size = 1) +

labs(title = "Risk", x = "log-transformed PFOA level", y = "OR(95%CI)",color = "age_group",fill = "age_group" )

#PFOS

model <-lrm(MCQ160N~rcs(log.PFOS,4)+age_group+LBXSUA+RIDAGEYR + RIAGENDR + RIDRETH1 + BMXBMI + DMDEDUC2 + DMDMARTL + INDFMPIR,data= cleaned_merge)

summary(model)

AGE <- Predict(model,

log.PFOS,

age_group = levels(cleaned_merge$ age_group),

fun = exp,

type = "predictions",

ref.zero = TRUE,

conf.int = 0.95)

summary(AGE)

ggplot() +

geom_line(data = AGE, aes(log.PFOS, yhat, color = age_group),

linetype = "solid", size = 1, alpha = 0.9) +

geom_ribbon(data = AGE,

aes(log.PFOS, ymin = lower, ymax = upper, fill = age_group),

alpha = 0.2) +

scale_color_manual(values = c("red", "#9Ec4be","#abd0f1", "#dce9f4"),

labels = c("20-39", "40-59","≥60")) +

scale_fill_manual(values = c("pink", "#9Ec4be", "#abd0f1", "#dce9f4"),

labels = c("20-39", "40-59","≥60")) +

theme_classic() +

geom_hline(yintercept = 1, linetype = 2, size = 1) +

labs(title = "Risk", x = "log-transformed PFOS level", y = "OR(95%CI)",color = "age_group",fill = "age_group" )

#PFHS

model <-lrm(MCQ160N~rcs(log.PFHxS,4)+age_group+LBXSUA+RIDAGEYR + RIAGENDR + RIDRETH1 + BMXBMI + DMDEDUC2 + DMDMARTL + INDFMPIR,data= cleaned_merge)

summary(model)

AGE <- Predict(model,

log.PFHxS,

age_group = levels(cleaned_merge$ age_group),

fun = exp,

type = "predictions",

ref.zero = TRUE,

conf.int = 0.95)

summary(AGE)

ggplot() +

geom_line(data = AGE, aes(log.PFHxS, yhat, color = age_group),

linetype = "solid", size = 1, alpha = 0.9) +

geom_ribbon(data = AGE,

aes(log.PFHxS, ymin = lower, ymax = upper, fill = age_group),

alpha = 0.2) +

scale_color_manual(values = c("red", "#9Ec4be","#abd0f1", "#dce9f4"),

labels = c("20-39", "40-59","≥60")) +

scale_fill_manual(values = c("pink", "#9Ec4be", "#abd0f1", "#dce9f4"),

labels = c("20-39", "40-59","≥60")) +

theme_classic() +

geom_hline(yintercept = 1, linetype = 2, size = 1) +

labs(title = "Risk", x = "log-transformed PFHxS level", y = "OR(95%CI)",color = "age_group",fill = "age_group" )

#PFNA

model <-lrm(MCQ160N~rcs(log.PFNA,4)+age_group+LBXSUA+RIDAGEYR + RIAGENDR + RIDRETH1 + BMXBMI + DMDEDUC2 + DMDMARTL + INDFMPIR,data= cleaned_merge)

summary(model)

AGE <- Predict(model,

log.PFNA,

age_group = levels(cleaned_merge$ age_group),

fun = exp,

type = "predictions",

ref.zero = TRUE,

conf.int = 0.95)

summary(AGE)

#使用 gplot 绘制图形

ggplot() +

geom_line(data = AGE, aes(log.PFNA, yhat, color = age_group),

linetype = "solid", size = 1, alpha = 0.9) +

geom_ribbon(data = AGE,

aes(log.PFNA, ymin = lower, ymax = upper, fill = age_group),

alpha = 0.2) +

scale_color_manual(values = c("red", "#9Ec4be","#abd0f1", "#dce9f4"),

labels = c("20-39", "40-59","≥60")) +

scale_fill_manual(values = c("pink", "#9Ec4be", "#abd0f1", "#dce9f4"),

labels = c("20-39", "40-59","≥60")) +

theme_classic() +

geom_hline(yintercept = 1, linetype = 2, size = 1) +

labs(title = "Risk", x = "log-transformed PFNA level", y = "OR(95%CI)",color = "age_group",fill = "age_group" )

#性别：

cleaned_merge$ RIAGENDR <- as.factor(cleaned_merge$ RIAGENDR)

# 使用lrm()函数拟合模型

dd <- datadist(cleaned_merge)

options(datadist = "dd")

#PFOA

model <-lrm(MCQ160N~rcs(log.PFOA,4)+LBXSUA+RIDAGEYR + RIAGENDR + RIDRETH1 + BMXBMI + DMDEDUC2 + DMDMARTL + INDFMPIR,data= cleaned_merge)

summary(model)

AGE <- Predict(model,log.PFOA,

RIAGENDR = levels(cleaned_merge$ RIAGENDR),

fun = exp,

type = "predictions",

ref.zero = TRUE,

conf.int = 0.95)

#使用 gplot 绘制图形

library(ggplot2)

ggplot() +

geom_line(data = AGE, aes(log.PFOA, yhat, color = RIAGENDR),

linetype = "solid", size = 1, alpha = 0.9) +

geom_ribbon(data = AGE,

aes(log.PFOA, ymin = lower, ymax = upper, fill = RIAGENDR),

alpha = 0.2) +

scale_color_manual(values = c("#43978F", "#9Ec4be", "#abd0f1", "#dce9f4"),

labels = c("Male", "Female")) +

scale_fill_manual(values = c("#43978F", "#9Ec4be", "#abd0f1", "#dce9f4"),

labels = c("Male", "Female")) +

theme_classic() +

geom_hline(yintercept = 1, linetype = 2, size = 1) +

labs(title = "Risk", x = "log-transformed PFOA level", y = "OR(95%CI)",color = "Sex",fill = "Sex" )

#PFOS

model <-lrm(MCQ160N~rcs(log.PFOS,4)+LBXSUA+RIDAGEYR + RIAGENDR + RIDRETH1 + BMXBMI + DMDEDUC2 + DMDMARTL + INDFMPIR,data= cleaned_merge)

summary(model)

AGE <- Predict(model,log.PFOS,

RIAGENDR = levels(cleaned_merge$ RIAGENDR),

fun = exp,

type = "predictions",

ref.zero = TRUE,

conf.int = 0.95)

#使用 gplot 绘制图形

library(ggplot2)

ggplot() +

geom_line(data = AGE, aes(log.PFOS, yhat, color = RIAGENDR),

linetype = "solid", size = 1, alpha = 0.9) +

geom_ribbon(data = AGE,

aes(log.PFOS, ymin = lower, ymax = upper, fill = RIAGENDR),

alpha = 0.2) +

scale_color_manual(values = c("#43978F", "#9Ec4be", "#abd0f1", "#dce9f4"),

labels = c("Male", "Female")) +

scale_fill_manual(values = c("#43978F", "#9Ec4be", "#abd0f1", "#dce9f4"),

labels = c("Male", "Female")) +

theme_classic() +

geom_hline(yintercept = 1, linetype = 2, size = 1) +

labs(title = "Risk", x = "log-transformed PFOS level", y = "OR(95%CI)",color = "Sex",fill = "Sex" )

#PFHxS

model <-lrm(MCQ160N~rcs(log.PFHxS,4)+LBXSUA+RIDAGEYR + RIAGENDR + RIDRETH1 + BMXBMI + DMDEDUC2 + DMDMARTL + INDFMPIR,data= cleaned_merge)

summary(model)

AGE <- Predict(model,log.PFHxS,

RIAGENDR = levels(cleaned_merge$ RIAGENDR),

fun = exp,

type = "predictions",

ref.zero = TRUE,

conf.int = 0.95)

#使用 gplot 绘制图形

library(ggplot2)

ggplot() +

geom_line(data = AGE, aes(log.PFHxS, yhat, color = RIAGENDR),

linetype = "solid", size = 1, alpha = 0.9) +

geom_ribbon(data = AGE,

aes(log.PFHxS, ymin = lower, ymax = upper, fill = RIAGENDR),

alpha = 0.2) +

scale_color_manual(values = c("#43978F", "#9Ec4be", "#abd0f1", "#dce9f4"),

labels = c("Male", "Female")) +

scale_fill_manual(values = c("#43978F", "#9Ec4be", "#abd0f1", "#dce9f4"),

labels = c("Male", "Female")) +

theme_classic() +

geom_hline(yintercept = 1, linetype = 2, size = 1) +

labs(title = "Risk", x = "log-transformed PFHxS level", y = "OR(95%CI)",color = "Sex",fill = "Sex" )

#PFNA

model <-lrm(MCQ160N~rcs(log.PFNA,4)+LBXSUA+RIDAGEYR + RIAGENDR + RIDRETH1 + BMXBMI + DMDEDUC2 + DMDMARTL + INDFMPIR,data= cleaned_merge)

summary(model)

AGE <- Predict(model,log.PFNA,

RIAGENDR = levels(cleaned_merge$ RIAGENDR),

fun = exp,

type = "predictions",

ref.zero = TRUE,

conf.int = 0.95)

#使用 gplot 绘制图形

library(ggplot2)

ggplot() +

geom_line(data = AGE, aes(log.PFNA, yhat, color = RIAGENDR),

linetype = "solid", size = 1, alpha = 0.9) +

geom_ribbon(data = AGE,

aes(log.PFNA, ymin = lower, ymax = upper, fill = RIAGENDR),

alpha = 0.2) +

scale_color_manual(values = c("#43978F", "#9Ec4be", "#abd0f1", "#dce9f4"),

labels = c("Male", "Female")) +

scale_fill_manual(values = c("#43978F", "#9Ec4be", "#abd0f1", "#dce9f4"),

labels = c("Male", "Female")) +

theme_classic() +

geom_hline(yintercept = 1, linetype = 2, size = 1) +

labs(title = "Risk", x = "log-transformed PFNA level", y = "OR(95%CI)",color = "Sex",fill = "Sex" )

#3.5.2 Mixture PFAS analysis#########################################################################################3

library(gWQS)

library(ggplot2)

library(knitr)

library(kableExtra)

library(reshape2)

library(dplyr)

CEP <- c("log.PFOA", "log.PFOS", "log.PFHxS", "log.PFNA")

cleaned_merge$ALQ120Q <- factor(cleaned_merge$ALQ120Q)

cleaned_merge$SMQ020 <- factor(cleaned_merge$SMQ020)

cleaned_merge$PAQ180 <- factor(cleaned_merge$PAQ180)

cleaned_merge$age_group

first_age_group_data <- cleaned_merge%>%

filter(age_group == "20-39")

model3_age1 <- gwqs(MCQ160N ~ wqs + ALQ120Q + SMQ020 + PAQ180,

mix_name = CEP,

data = first_age_group_data,

q = 4,

validation = 0.6,

b = 10,

b1_pos = TRUE,

rh = 10,

family = "binomial",

covariables = ~ LBXSUA+RIDAGEYR + RIAGENDR + RIDRETH1 + BMXBMI + DMDEDUC2 + DMDMARTL + INDFMPIR)

first_age_group_data2 <- cleaned_merge %>%

filter(age_group == "40-59")

model3_age2 <- gwqs(MCQ160N ~ wqs + ALQ120Q + SMQ020 + PAQ180,

mix_name = CEP,

data = first_age_group_data2,

q = 4,

validation = 0.6,

b = 10,

b1_pos = TRUE,

rh = 10,

family = "binomial",

covariables = ~ LBXSUA+RIDAGEYR + RIAGENDR + RIDRETH1 + BMXBMI + DMDEDUC2 + DMDMARTL + INDFMPIR)

first_age_group_data3 <- cleaned_merge %>%

filter(age_group == "≥60")

model3_age3 <- gwqs(MCQ160N ~ wqs + ALQ120Q + SMQ020 + PAQ180,

mix_name = CEP,

data = first_age_group_data3,

q = 4,

validation = 0.6,

b = 10,

b1_pos = TRUE,

rh = 10,

family = "binomial",

covariables = ~ LBXSUA+RIDAGEYR + RIAGENDR + RIDRETH1 + BMXBMI + DMDEDUC2 + DMDMARTL + INDFMPIR)

summary(model3_age1)

summary(model3_age2)

summary(model3_age3)

#性别

data1 <- cleaned_merge%>%

filter(RIAGENDR == 1)

model3_SEX1 <- gwqs(MCQ160N ~ wqs + ALQ120Q + SMQ020 + PAQ180,

mix_name = CEP,

data = data1,

q = 4,

validation = 0.6,

b = 10,

b1_pos = TRUE,

rh = 10,

family = "binomial",

covariables = ~ LBXSUA+RIDAGEYR + RIAGENDR + RIDRETH1 + BMXBMI + DMDEDUC2 + DMDMARTL + INDFMPIR)

data2 <- cleaned_merge%>%

filter(RIAGENDR == 2)

model3_SEX2 <- gwqs(MCQ160N ~ wqs + ALQ120Q + SMQ020 + PAQ180,

mix_name = CEP,

data = data2,

q = 4,

validation = 0.6,

b = 10,

b1_pos = TRUE,

rh = 10,

family = "binomial",

covariables = ~ LBXSUA+RIDAGEYR + RIAGENDR + RIDRETH1 + BMXBMI + DMDEDUC2 + DMDMARTL + INDFMPIR)

summary(model3_SEX1)

summary(model3_SEX2)

gwqs_barplot(model3_age1)

gwqs_barplot(model3_age2)

gwqs_barplot(model3_age3)

gwqs_barplot(model3_SEX1)

gwqs_barplot(model3_SEX2)

#3.6 Sensitivity analysis##########################################################################################################3

pfas_vars <- c("log.PFOA", "log.PFOS", "log.PFHxS", "log.PFNA") # 假设这些是PFAS含量的变量名

pfas_99th_percentiles <- apply(cleaned_merge[, pfas_vars], 2, function(x) quantile(x, 0.99))

merge_clear_filtered <- cleaned_merge[apply(cleaned_merge[, pfas_vars], 1, function(row) all(row <= pfas_99th_percentiles)), ]

#Model1: 不控制协变量

#PFOA

model_PFOA_0I <- glm(MCQ160N ~ log.PFOA, data =merge_clear_filtered, family = "binomial")

#PFOS

model_PFOS_0I <-glm(MCQ160N ~ log.PFOS, data =merge_clear_filtered, family = "binomial")

#PFHS

model_PFHS_0I <-glm(MCQ160N ~ log.PFHxS, data =merge_clear_filtered, family = "binomial")

#PFNA

model_PFNA_0I <-glm(MCQ160N ~ log.PFNA, data =merge_clear_filtered, family = "binomial")

merge_clear_filtered$RIAGENDR <- as.factor(merge_clear_filtered$RIAGENDR)

#Model2: 协变量 + RIDAGEYR + RIAGENDR

#PFOA

model_PFOA_2I <- glm(MCQ160N~ log.PFOA + RIDAGEYR + RIAGENDR, data =merge_clear_filtered , family = "binomial")

#PFOS

model_PFOS_2I <-glm(MCQ160N~ log.PFOS + RIDAGEYR + RIAGENDR, data =merge_clear_filtered, family = "binomial")

#PFHS

model_PFHS_2I <-glm(MCQ160N~ log.PFHxS + RIDAGEYR + RIAGENDR, data =merge_clear_filtered, family = "binomial")

#PFNA

model_PFNA_2I <-glm(MCQ160N~ log.PFNA + RIDAGEYR + RIAGENDR, data =merge_clear_filtered, family = "binomial")

##Model3: 控制上述全部协变量

#PFOA

model_PFOA_7I <- glm(MCQ160N ~ log.PFOA + LBXSUA+RIDAGEYR + RIAGENDR+BMXBMI+RIDRETH1+DMDEDUC2+DMDMARTL+INDFMPIR, data =merge_clear_filtered, family = "binomial")

#PFOS

model_PFOS_7I <-glm(MCQ160N ~ log.PFOS + LBXSUA+RIDAGEYR + RIAGENDR+BMXBMI+RIDRETH1+DMDEDUC2+DMDMARTL+INDFMPIR, data =merge_clear_filtered, family = "binomial")

#PFHS

model_PFHS_7I <-glm(MCQ160N ~ log.PFHxS + LBXSUA+RIDAGEYR + RIAGENDR+BMXBMI+RIDRETH1+DMDEDUC2+DMDMARTL+INDFMPIR, data =merge_clear_filtered, family = "binomial")

#PFNA

model_PFNA_7I <-glm(MCQ160N ~ log.PFNA + LBXSUA+RIDAGEYR + RIAGENDR+BMXBMI+RIDRETH1+DMDEDUC2+DMDMARTL+INDFMPIR, data =merge_clear_filtered, family = "binomial")

summary(model_PFOA_0I)

summary(model_PFOS_0I)

summary(model_PFHS_0I)

summary(model_PFNA_0I)

summary(model_PFOA_2I)

summary(model_PFOS_2I)

summary(model_PFHS_2I)

summary(model_PFNA_2I)

summary(model_PFOA_7I)

summary(model_PFOS_7I)

summary(model_PFHS_7I)

summary(model_PFNA_7I)

model1_0I<- gwqs(MCQ160N ~ wqs+ALQ120Q+SMQ020+PAQ180, mix_name = CEP, data =merge_clear_filtered,q = 4,

validation = 0.6, b = 10, b1_pos = TRUE, rh = 10, family = "binomial")

model2_2I<- gwqs(MCQ160N ~ wqs+ALQ120Q+SMQ020+PAQ180, mix_name = CEP, data =merge_clear_filtered, q = 4,

validation = 0.6, b = 10, b1_pos = TRUE, rh = 10, family = "binomial",

covariables = ~ RIDAGEYR + RIAGEND)

model3_7I<- gwqs(MCQ160N~ wqs+ALQ120Q+SMQ020+PAQ180, mix_name = CEP, data =merge_clear_filtered, q = 4,

validation = 0.6, b = 10, b1_pos = TRUE, rh = 10, family = "binomial",

covariables = ~ LBXSUA+RIDAGEYR + RIAGENDR + RIDRETH1 + BMXBMI + DMDEDUC2 + DMDMARTL + INDFMPIR)

summary(model1_0I)

summary(model2_2I)

summary(model3_7I)

gwqs_barplot(model1_0I)

gwqs_barplot(model2_2I)

gwqs_barplot(model3_7I)

#敏感性分析补充：

# 定义不同周期对应的最低可测浓度

min_detect_conc <- data.frame(

cycle = c("2007-2008", "2009-2010", "2011-2012", "2013-2014", "2015-2016", "2017-2018"),

log_PFOA = c(0.1, 0.1, 0.1, 0.1, 0.1, 0.1),

log_PFOS = c(0.2, 0.2, 0.2, 0.1, 0.1, 0.1),

log_PFHxS = c(0.1, 0.1, 0.1, 0.1, 0.1, 0.1),

log_PFNA = c(0.08, 0.08, 0.08, 0.1, 0.1, 0.1)

)

cleaned_merge <- merge(cleaned_merge, min_detect_conc, by = "cycle")

set.seed(123) # 设置随机种子以便结果可复现

cleaned_merge$log_PFOA_new <- ifelse(cleaned_merge$log.PFOA == cleaned_merge$log_PFOA,

runif(nrow(cleaned_merge), min = 0, max = cleaned_merge$log_PFOA),

cleaned_merge$log.PFOA)

cleaned_merge$log_PFOS_new <- ifelse(cleaned_merge$log.PFOS == cleaned_merge$log_PFOS,

runif(nrow(cleaned_merge), min = 0, max = cleaned_merge$log_PFOS),

cleaned_merge$log.PFOS)

cleaned_merge$log_PFHxS_new <- ifelse(cleaned_merge$log.PFHxS == cleaned_merge$log_PFHxS,

runif(nrow(cleaned_merge), min = 0, max = cleaned_merge$log_PFHxS),

cleaned_merge$log.PFHxS)

cleaned_merge$log_PFNA_new <- ifelse(cleaned_merge$log.PFNA == cleaned_merge$log_PFNA,

runif(nrow(cleaned_merge), min = 0, max = cleaned_merge$log_PFNA),

cleaned_merge$log.PFNA)

#Model1: 不控制协变量

#PFOA

a1 <- glm(MCQ160N ~ log_PFOA_new, data =cleaned_merge, family = "binomial")

summary(a1)

#PFOS

a2<- glm(MCQ160N ~ log_PFOS_new, data =cleaned_merge, family = "binomial")

summary(a2)

#PFHS

a3 <- glm(MCQ160N ~ log_PFHxS_new, data =cleaned_merge, family = "binomial")

summary(a3)

#PFNA

a4 <- glm(MCQ160N ~ log_PFNA_new, data =cleaned_merge, family = "binomial")

summary(a4)

cleaned_merge$RIAGENDR <- as.factor(cleaned_merge$RIAGENDR)

#Model2: 协变量 + RIDAGEYR + RIAGENDR

#PFOA

b1 <- glm(MCQ160N~ log_PFOA_new + RIDAGEYR + RIAGENDR, data =cleaned_merge , family = "binomial")

summary(b1)

#PFOS

b2 <-glm(MCQ160N~ log_PFOS_new + RIDAGEYR + RIAGENDR, data =cleaned_merge, family = "binomial")

summary(b2)

#PFHS

b3 <-glm(MCQ160N~ log_PFHxS_new + RIDAGEYR + RIAGENDR, data =cleaned_merge, family = "binomial")

summary(b3)

#PFNA

b4 <-glm(MCQ160N~ log_PFNA_new + RIDAGEYR + RIAGENDR, data =cleaned_merge, family = "binomial")

summary(b4)

##Model3: 控制上述全部协变量

#PFOA

C1 <- glm(MCQ160N ~ log_PFOA_new + LBXSUA+RIDAGEYR + RIAGENDR+BMXBMI+RIDRETH1+DMDEDUC2+DMDMARTL+INDFMPIR, data =cleaned_merge, family = "binomial")

summary(C1)

#PFOS

C2 <-glm(MCQ160N ~ log_PFOS_new + LBXSUA+RIDAGEYR + RIAGENDR+BMXBMI+RIDRETH1+DMDEDUC2+DMDMARTL+INDFMPIR, data =cleaned_merge, family = "binomial")

summary(C2)

#PFHS

C3 <-glm(MCQ160N ~ log_PFHxS_new + LBXSUA+RIDAGEYR + RIAGENDR+BMXBMI+RIDRETH1+DMDEDUC2+DMDMARTL+INDFMPIR, data =cleaned_merge, family = "binomial")

summary(C3)

#PFNA

C4 <-glm(MCQ160N ~ log_PFNA_new + LBXSUA+RIDAGEYR + RIAGENDR+BMXBMI+RIDRETH1+DMDEDUC2+DMDMARTL+INDFMPIR, data =cleaned_merge, family = "binomial")

summary(C4)

#WQS

CEP <- c("log_PFOA_new", "log_PFOS_new", "log_PFHxS_new", "log_PFNA_new")

WQS1<- gwqs(MCQ160N ~ wqs+ALQ120Q+SMQ020+PAQ180, mix_name = CEP, data =cleaned_merge,q = 4,

validation = 0.6, b = 10, b1_pos = TRUE, rh = 10, family = "binomial")

WQS2<- gwqs(MCQ160N ~ wqs+ALQ120Q+SMQ020+PAQ180, mix_name = CEP, data =cleaned_merge, q = 4,

validation = 0.6, b = 10, b1_pos = TRUE, rh = 10, family = "binomial",

covariables = ~ RIDAGEYR + RIAGEND)

WQS3<- gwqs(MCQ160N~ wqs+ALQ120Q+SMQ020+PAQ180, mix_name = CEP, data =cleaned_merge, q = 4,

validation = 0.6, b = 10, b1_pos = TRUE, rh = 10, family = "binomial",

covariables = ~ LBXSUA+RIDAGEYR + RIAGENDR + RIDRETH1 + BMXBMI + DMDEDUC2 + DMDMARTL + INDFMPIR)

summary(WQS1)

summary(WQS2)

summary(WQS3)

gwqs_barplot(WQS1)

gwqs_barplot(WQS2)

gwqs_barplot(WQS3)

#3.7 Analysis of intermediary effect###################################################

library(mediation)

#log.PFOA, , ,

model.m <- lm(LBXSUA ~ log.PFOA, cleaned_merge)

model.y <- lm(MCQ160N ~ log.PFOA + LBXSUA, cleaned_merge)

med <- mediate(model.m,model.y,treat="log.PFOA",mediator = "LBXSUA", boot = TRUE, sims = 5000)

summary(med)

#log.PFOS

model.m <- lm(LBXSUA ~ log.PFOS, cleaned_merge)

model.y <- lm(MCQ160N ~ log.PFOS + LBXSUA, cleaned_merge)

med <- mediate(model.m,model.y,treat="log.PFOS",mediator = "LBXSUA", boot = TRUE, sims = 5000)

summary(med)

#log.PFHS

model.m <- lm(LBXSUA ~ log.PFHS, cleaned_merge)

model.y <- lm(MCQ160N ~ log.PFHS + LBXSUA, cleaned_merge)

med <- mediate(model.m,model.y,treat="log.PFHS",mediator = "LBXSUA", boot = TRUE, sims = 5000)

summary(med)

#log.PFNA

model.m <- lm(LBXSUA ~ log.PFNA, cleaned_merge)

model.y <- lm(MCQ160N ~ log.PFNA + LBXSUA, cleaned_merge)

med <- mediate(model.m,model.y,treat="log.PFNA",mediator = "LBXSUA", boot = TRUE, sims = 5000)

summary(med)
